# Safety of the novel oral poliovirus vaccine type 2 (nOPV2) in infants and young children aged 1 to <5 years and lot-to-lot consistency of the immune response to nOPV2 in infants in The Gambia: a phase 3, double-blind, randomised controlled trial

**DOI:** 10.1016/S0140-6736(23)02844-1

**Published:** 2024-03-23

**Authors:** Magnus Ochoge, Ahmed Cherno Futa, Ama Umesi, Lucy Affleck, Larry Kotei, Baboucarr Daffeh, Ebrima Saidy-Jah, Anna Njie, Oluwafemi Oyadiran, Bassey Edem, Musa Jallow, Edrissa Jallow, Simon A Donkor, Erman Tritama, Talha Abid, Kathryn A V Jones, Bernardo A Mainou, John O Konz, Alan Fix, Chris Gast, Ed Clarke

**Affiliations:** a MRC Unit The Gambia at the London School of Hygiene and Tropical Medicine, Banjul, The Gambia; b Research and Development Division, PT Bio Farma, Bandung, Indonesia; c Division of Viral Diseases, National Center for Immunization and Respiratory Diseases, US Centers for Disease Control and Prevention, Atlanta, GA, USA; d Center for Vaccine Innovation and Access, PATH, Seattle, WA, USA

## Abstract

**Background:**

Novel oral poliovirus vaccine type 2 (nOPV2) has been engineered to improve the genetic stability of Sabin oral poliovirus vaccine (OPV) and reduce the emergence of circulating vaccine-derived polioviruses. This trial aimed to provide key safety and immunogenicity data required for nOPV2 licensure and WHO prequalification.

**Methods:**

This phase 3 trial recruited infants aged 18 to <52 weeks and young children aged 1 to <5 years in The Gambia. Infants randomly assigned to receive one or two doses of one of three lots of nOPV2 or one lot of bivalent OPV (bOPV). Young children were randomised to receive two doses of nOPV2 lot 1 or bOPV. The primary immunogenicity objective was to assess lot-to-lot equivalence of the three nOPV2 lots based on one-dose type 2 poliovirus neutralising antibody seroconversion rates in infants. Equivalence was declared if the 95% CI for the three pairwise rate differences was within the –10% to 10% equivalence margin. Tolerability and safety were assessed based on the rates of solicited adverse events to 7 days, unsolicited adverse events to 28 days, and serious adverse events to 3 months post-dose. Stool poliovirus excretion was examined. The trial was registered as PACTR202010705577776 and is completed.

**Findings:**

Between February and October, 2021, 2345 infants and 600 young children were vaccinated. 2272 (96·9%) were eligible for inclusion in the post-dose one per-protocol population. Seroconversion rates ranged from 48·9% to 49·2% across the three lots. The minimum lower bound of the 95% CIs for the pairwise differences in seroconversion rates between lots was –5·8%. The maximum upper bound was 5·4%. Equivalence was therefore shown. Of those seronegative at baseline, 143 (85·6%) of 167 (95% CI 79·4–90·6) infants and 54 (83·1%) of 65 (71·7–91·2) young children seroconverted over the two-dose nOPV2 schedule. The post-two-dose seroprotection rates, including participants who were both seronegative and seropositive at baseline, were 604 (92·9%) of 650 (95% CI 90·7–94·8) in infants and 276 (95·5%) of 289 (92·4–97·6) in young children. No safety concerns were identified. 7 days post-dose one, 78 (41·7%) of 187 (95% CI 34·6–49·1) infants were excreting the type 2 poliovirus.

**Interpretation:**

nOPV2 was immunogenic and safe in infants and young children in The Gambia. The data support the licensure and WHO prequalification of nOPV2.

**Funding:**

Bill & Melinda Gates Foundation.

## Introduction

Polio eradication is within reach, although hurdles remain. Wild polioviruses type 2 and type 3 were certified as eradicated in 2015 and 2019, respectively. Only Pakistan and Afghanistan remain endemic for wild poliovirus type 1 and reported a total of 11 cases of paralytic disease in the 12 months leading to November, 2023.^1^

Oral polio vaccines (OPVs) remain crucial to the Global Polio Eradication Initiative's endgame strategy.^2^ The vaccines not only generate systemic antibodies, but also mucosal immunity, preventing prolonged excretion of the virus and community transmission.^3^ Their disadvantage is the emergence of circulating vaccine-derived polioviruses (cVDPVs) that have lost attenuating mutations, thus becoming a source of paralytic disease; outbreaks occur in populations with low vaccine coverage.^2^ In April, 2016, a switch from trivalent to bivalent OPV (bOPV), containing only the Sabin type 1 and type 3 strains, was made globally. The switch aimed to reduce the occurrence of cVDPVs, 85% of which were attributable to the type 2 virus at the time. However, it also resulted in a marked decrease in type 2 mucosal immunity globally.^4^, ^5^ Thus, although there were less than 100 cVDPV type 2 (cVDPV2) cases in two countries in 2017, there were more than 1000 cases in 24 countries by 2020.^6^ The detection of cVDPV2 in Europe, the USA, and southeast Asia further emphasises the global nature of the threat posed.^6^

**Research in context**

**Evidence before this study**

A PubMed search to identify articles published before Sept 1, 2023 from database inception was conducted using the following search terms with appropriate Boolean operators: “oral poliovirus vaccine”, “vaccine derived poliovirus”, “campaign”, “meta-analysis”, “systematic review”, “randomized controlled trial”, “clinical trial”, “immunogenicity”, “safety”, “mucosal immunity”. Novel oral poliovirus vaccine type 2 (nOPV2) has been engineered to increase the genetic stability of type 2 Sabin oral poliovirus vaccine (OPV) and hence to reduce the risk of paralytic outbreaks of circulating vaccine-derived poliovirus type 2 (cVDPV2). In clinical trials done in adults (ie, those aged 18–50 years) in Belgium, in infants (ie, those aged 18–22 weeks) and children (ie, those aged 1–4 years) in Panama, and newborns in Bangladesh, the vaccine has been shown to be well tolerated and safe. In Panama, 86–94% of infants and children had an immune response following one dose of nOPV2 and at least 98% had an immune response following two doses of the vaccine. The immunogenicity of the vaccine was comparable with that of Sabin monovalent OPV type 2 (mOPV2) in historical controls. Trials of nOPV2 produced evidence of mucosal immunity generation and confirmed a reduced risk of reversion to neurovirulence than type 2 Sabin OPV. On the basis of these data, nOPV2 received Emergency Use Listing from WHO in November, 2020. More than 800 million doses of the vaccine have subsequently been administered across 28 countries. Analysis of data collected following this roll-out suggest the vaccine is safe and has a comparable effect on cVDPV2 outbreaks as mOPV2 while being associated with considerably fewer genetic reversion events.

**Added value of this study**

This clinical trial is the first of nOPV2 in Africa, which has had the greatest burden of cVDPV2 since trivalent OPV withdrawal. In this population, equivalence of three manufacturing lots of nOPV2 was shown based on single dose seroconversion rates in infants previously primed with a single inactivated poliovirus vaccine dose. The lots included levels of genetic heterogeneity in nOPV2 strains expected during routine manufacture. Around two-thirds of infants and young children who were poliovirus type 2 serum neutralising antibody seronegative at baseline seroconverted following one dose of nOPV2, increasing to more than 80% following two doses. Baseline type 2 seroprotection rates, of around 75% in both age groups, increased to 92% and 96% following two doses of nOPV2. The rate of solicited and unsolicited adverse events after nOPV2 was comparable with the rate after bivalent OPV. There were no severe or serious adverse events after nOPV2 that were considered to be related to the vaccines. Only 5% of infants tested were shedding type 2 virus in their stool after 28 days with none shedding type 2 virus 84 days after a single dose of nOPV2.

**Implications of all the available evidence**

nOPV2 is safe and immunogenic in infants and young children in The Gambia—having a comparable safety and immunogenicity profile to the well established Sabin OPV strains. Data collected in clinical trials, and through deployment of the vaccine under the Emergency Use Listing, confirm the improved genetic stability of the vaccine. The effect of nOPV2 in controlling cVDPV2 outbreaks is comparable with that of mOPV2 while the former is less likely to lose attenuation and appears less likely to seed further genetic reversion events. The data support the licensure and WHO prequalification of nOPV2 as a vital tool in the polio eradication endgame strategy.

Given the need to block transmission, Sabin monovalent OPV type 2 (mOPV2) immunisation campaigns have been the predominant response to cVDPV2 outbreaks. Consequently, although earlier cVDPV2 outbreaks were seeded from trivalent oral poliovirus vaccines used before the switch, sequencing data confirm that recent outbreaks have arisen from mOPV2 administered in such campaigns.^7^

The novel OPV type 2 (nOPV2) strain has been engineered, through the introduction of a series of genetic modifications, to improve the genetic stability of the Sabin type 2 OPV and thus reduce cVDPV2 emergence.^8^ On the basis of supportive pre-clinical data, the vaccine was tested in phase 1 and phase 2 trials in adults (ie, those aged 18–50 years) in Belgium, in phase 2 trials in children aged 1–4 years in Panama, in phase 2 trials in infants aged 18–22 weeks in Panama, and in phase 2 trials in newborns in Bangladesh.^8^, ^9^, ^10^, ^11^, ^12^ In all age groups, nOPV2 was well tolerated and safe. In adults, children, and infants, serological responses to nOPV2 were similar to those generated in historic mOPV2 controls.^9^, ^10^, ^11^ The vaccine was immunogenic in newborns.^12^ Reduced shedding following second doses relative to first doses indicated generation of mucosal immune responses.^13^ Neurovirulence testing of shed virus in mice, supported by next-generation sequencing, confirmed a reduced risk of reversion to virulence following nOPV2 compared with mOPV2 immunisation.^14^

In November, 2020, based on these data, in the context of the rapid increase in cVDPV2 outbreaks and their status as a Public Health Emergency of International Concern, nOPV2 was the first vaccine to be given Emergency Use Listing by WHO.^15^ The vaccine was initially used as part of a cVDPV2 outbreak response in Nigeria in March, 2021, and more than 800 million doses of the vaccine have subsequently been administered in campaigns across 28 countries.^16^

This phase 3 trial aimed to generate the data required for nOPV2 licensure and for its prequalification by WHO. Reflecting this aim, it had two primary objectives. First, to expand the safety and tolerability data on nOPV2 with data from infants aged 18 to <52 weeks and young children aged 1 to <5 years in The Gambia. Second, to assess the consistency of the immune responses to three consecutive, genetically heterogeneous, manufacturing lots of nOPV2 in infants. Secondary immunogenicity objectives were to determine the combined immunogenicity of a first and second dose of the three lots of nOPV2 in infants and of a first and second dose of a single lot of nOPV2 in young children. Poliovirus type 2 shedding in the stool was examined in a randomly selected subset of infants.

## Methods

### Study design and participants

This was a single-centre, double-blind, randomised, controlled, phase 3 trial. It was conducted at the Medical Research Council Unit The Gambia at the London School of Hygiene and Tropical Medicine. Participants were recruited through clinical trial facilities in three government health centres (Brikama, Banjulinding, and Fajikunda) in the western region of The Gambia. Healthy infants, who had received at least three doses of bOPV and a single dose of the inactivated poliovirus vaccine at least one month before randomisation, were eligible to join the study from aged 18 weeks until the day before they reached age 52 weeks. Healthy young children, who had received at least one dose of a type 2 poliovirus vaccine (trivalent oral poliovirus vaccine or inactivated poliovirus vaccine), at least one month before randomisation, were eligible to join the study from 1 to 5 years of age (appendix pp 2–5). Parents provided written informed consent.

The study was conducted in accordance with the Declaration of Helsinki and Good Clinical Practice guidelines. Approval was obtained from The Gambia Government and MRC joint ethics committee (LEO22052), the London School of Hygiene and Tropical Medicine Research Ethics Committee, the WIRB-Copernicus Group Institutional Review Board, and The Gambian Medicines Control Agency. The trial was registered with the Pan-African Clinical Trials Registry (PACTR202010705577776).

### Randomisation and masking

Eligible infants were randomly assigned 2:2:2:1 to receive one of three lots of nOPV2 (670 infants per group) or bOPV (335 infants total). In each of the three nOPV2 groups, 224 infants were randomly assigned to receive a second dose of the same nOPV2 lot, 28 days after the first. 112 infants in the bOPV group were randomly assigned to receive a second dose of bOPV in the same way. Eligible young children were randomly assigned 1:1 to receive two doses of either nOPV2 lot 1 or bOPV at an interval of 28 days. Randomisation sequences were generated by an independent statistician using permuted block sizes. Vaccine assignment was undertaken using a web-based randomisation system. Only unmasked nurses who were responsible for vaccine preparation and administration were aware of the vaccine assigned. Parents and all other staff were masked.

## Procedures

Following confirmation of eligibility, infants and young children received their first vaccine dose on day 0 (appendix p 6). Infants in the one-dose cohort had follow-up visits 28 days and 84 days post-dose. Serum samples were collected from infants in the one-dose group before vaccination and 28 days post-dose. Stool samples were collected from a randomly selected subset of infants in the one-dose group before vaccination and 7 days, 28 days, and 84 days post-dose. In the two-dose groups, the second vaccine dose was given 28 days after the first. Further follow-up visits in the two-dose groups were conducted 28 days after the second vaccine dose. Infants in the two-dose group and all young children had an end of study visit 112 days after the first vaccination. Serum samples were collected in the two-dose group before and 28 days after each vaccine dose.

A single 0·1 mL (two drop) dose of nOPV2 (PT Biofarma, Bandung, Indonesia) contains at least 10^5·0^ 50% cell culture infectious dose (CCID_50_) of the nOPV2 strain. The batch numbers for lots 1, 2, and 3 were 2220720, 2220820, and 2220920 respectively. 50-dose vials were used in the trial. Details of the genetic heterogeneity of the three lots at key loci, based on next-generation sequencing, are provided in the appendix (p 7). A single 0·1 mL (two drop) dose of bOPV (PT Biofarma) contains at least 10^6.0^ CCID_50_ of Sabin type 1 poliovirus and at least 10^5.8^ of Sabin type 3 poliovirus. 20-dose vials with a batch number of 2045119 were used in the trial.

Venous blood samples, of 3·0 mL, were collected at the specified timepoints. Serum was stored below –70°C before immunogenicity testing. Serotype-specific poliovirus serum neutralising antibodies (SNAs) were measured using the WHO standard microneutralisation assay (WHO EPI Gen 93.9).^17^ The lower limit of quantification for SNA titres in infants and young children was a log_2_ reciprocal titre of 2·5. For infants, the upper limit of quantification was a log_2_ reciprocal titre of 18·5. For young children the upper limit of quantification was a log_2_ reciprocal titre of 10·5. Stool samples were collected at the specified timepoints and stored frozen below –70°C before testing. Serotype-specific polioviruses were detected in the stool using a validated real-time RT-PCR assay.^18^ Laboratory analysis was conducted at the US Centers for Disease Control and Prevention (appendix p 8).

Infants in the two-dose groups and all young children had solicited adverse events data collected in clinic on the day of vaccination, through home visits conducted for 6 days, and at a clinic visit 7 days post-dose. Solicited adverse events were collected in this way after the first and second vaccine dose. Systemic solicited adverse events were fever (≥37·5°C), vomiting, diarrhoea, irritability, decreased feeding, and decreased activity.

In all infants and young children, unsolicited adverse events were collected from the day of the first vaccination until 28 days after the last vaccine dose. Serious adverse events were collected from the day of first vaccination until the end of study visit. Solicited and unsolicited adverse events were graded for severity from grade 1 (mild) to grade 4 (potentially life threatening) according to the Division of AIDS Tables (version 2.1; appendix pp 9–10). Any adverse event resulting in death was defined as grade 5. Unsolicited adverse events were classified according to the Medical Dictionary of Regulatory Activities (version 25.0) and were defined as related or unrelated to the study intervention. A Data Safety Monitoring Board reviewed accumulating safety data at intervals during trial conduct.

### Outcomes

The primary immunogenicity outcome, for the assessment of lot-to-lot consistency, was the proportion of infants undergoing poliovirus type 2 seroconversion 28 days after a single dose of nOPV2 vaccine. Seroconversion was defined as either a change from seronegative (ie, a reciprocal SNA titre of <8 [<3 log_2_]) at baseline to seropositive (ie, a reciprocal SNA titre of >8 [≥3 log_2_]) 28 days post-dose, or a minimum four-fold rise in SNA titres (difference of 2 log_2_) from baseline to 28 days post-dose in those who were already seropositive. The analysis in seropositive participants only included those who had a baseline titre sufficiently below the upper limit of quantification of the assay to allow a four-fold rise to be measured. As secondary immunogenicity outcomes, lot-to-lot consistency was also assessed based on seroprotection rates, defined as the proportion of participants with a reciprocal SNA titre of 8 or higher and geometric mean SNA titres. Additional secondary immunogenicity outcomes were the proportion of young children undergoing poliovirus type 2 seroconversion following a single nOPV2 vaccine dose, seroprotection rates, and median and geometric mean SNA titres at baseline and 28 days following a single dose of nOPV2 in infants and children and the seroconversion and seroprotection rates, and median and geometric mean SNA titres following a second dose of nOPV2 in young children and a subset of infants. The young children provided additional confirmation of immunogenicity in this demographic subgroup, which had not been studied in phase 2 trials with nOPV2 of the current potency. Viral shedding outcomes, collected in a randomly selected cohort of infants in the one-dose group, were the proportion of infants shedding type 2 poliovirus overall and at each collection timepoint and the time to cessation of shedding.

The primary tolerability and safety outcomes were the frequency of solicited adverse events during the 7 days post-dose (in young children and a subset of infants receiving 2 doses), of unsolicited adverse events from the day of the first vaccine dose until 28 days after the last vaccine dose, and of serious adverse events throughout the 3 months after the last study dose.

### Statistical analysis

A sample size of 670 infants per nOPV2 group was calculated to provide at least 90% power to show equivalence of the three manufacturing lots based on seroconversion rates, using an equivalence interval of –10% to 10% (appendix pp 11–18). The recruitment of a further 335 infants to receive bOPV was designed to provide sufficient power to detect an excess of adverse events in the 2010 infants who received nOPV2, compared with those who received bOPV (appendix p 13). A sample size of 300 young children in the nOPV2 group was chosen to detect uncommon events in this age group.

To show lot-to-lot equivalence, two-sided 95% Miettinen and Nurminen CIs for the differences in seroconversion rates between pairs of each of the three nOPV2 lots were calculated.^19^ Lot-to-lot equivalence was declared if the CI for each pairwise comparison was contained within the –10% to 10% equivalence margin. Exact Clopper-Pearson 95% CIs were calculated for seroconversion, seroprotection, and adverse event rates. Median log_2_ SNA titres and associated bootstrap 95% CIs were calculated. Geometric mean titres and 95% CIs were calculated based on survival methods, allowing for censoring at the lower limit of quantification and upper limit of quantification. Safety event rates were compared using a two-sided Fisher's exact test. Rates of type 2 poliovirus shedding with exact Clopper-Pearson two-sided 95% CIs were calculated. Details of the analysis populations are provided in the appendix (p 20).

### Role of the funding source

The Bill & Melinda Gates Foundation funded PATH to conduct this study but had no role in data collection, analysis, interpretation, manuscript writing, or the decision to submit the manuscript for publication. PATH was involved in the designs, conduct, analysis, and reporting of the study.

## Results

**Figure 1:**
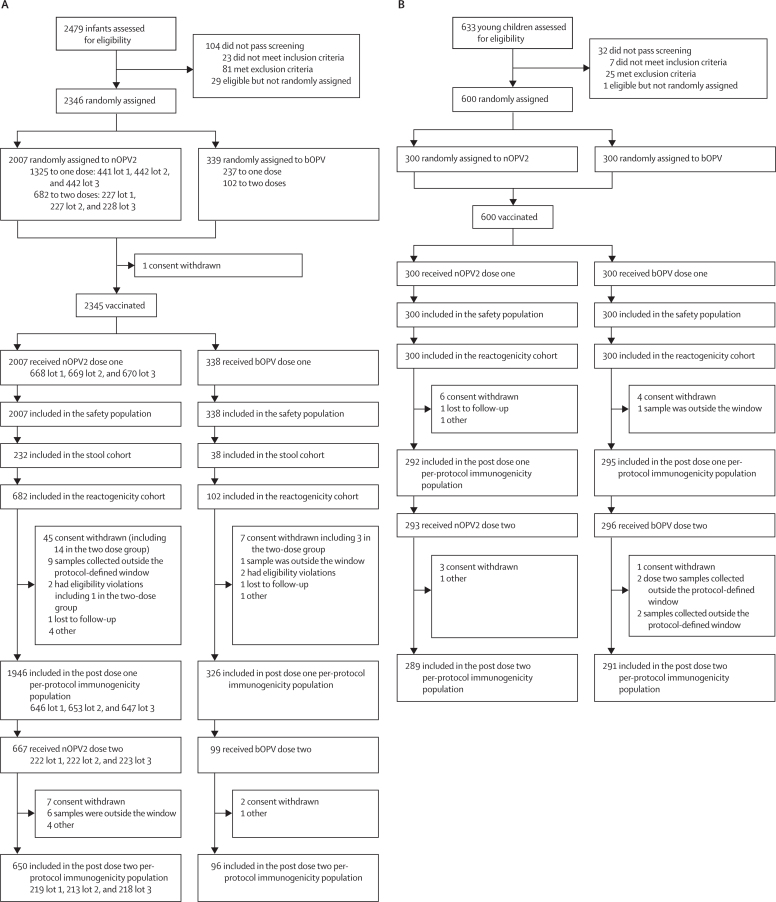
Trial profile (A) Infants trial profile. (B) Young children trial profile. All young children randomly assigned to nOPV2 received lot 1 of the vaccine. bOPV=bivalent oral poliovirus vaccine. nOPV2=novel oral poliovirus vaccine type 2.

Between February and October, 2021, 2479 infants were assessed for study eligibility, 2346 (94·6%) were randomly assigned and 2345 were vaccinated (figure 1). 2272 infants (96·9%) were eligible for inclusion in the post-dose one per-protocol population and 746 infants (95·2%) in the post-dose two per-protocol population. All vaccinated infants were included in the post-dose one safety population, while 766 (97·7%) were included in the post-dose two safety population.

Between the same dates, 633 young children were assessed for eligibility, and 600 were randomised and vaccinated (figure 1). Overall, 587 young children (97·8%) were eligible for inclusion in the post-dose one per-protocol population and 580 (96·7%) in the post-dose two per-protocol population. All young children were included in the post-dose one safety population, while 589 (98·2%) were included in the post-dose two safety population.

There were no clinically meaningful differences in the anthropometric characteristics of infants or young children enrolled into the nOPV2 and bOPV groups (table 1), or between infants receiving different lots of nOPV2 (appendix p 21). 1154 (49·2%) of 2345 infants and 296 (49·3%) of 600 young children were female. Between 5·0% and 9·3% of infants and children were at least moderately malnourished (weight for height Z score of <–2 SD). Previous polio vaccination history was similar between groups and reflected the eligibility criteria for the trial (appendix p 22).

**Table 1:** Baseline demographic and anthropometric characteristics in infants and young children enrolled in the safety population

		**Infants, one dose groups**		**Infants, two dose groups**		**Young children**	
		nOPV2 (n=1325)	bOPV (n=236)	nOPV2 (n=682)	bOPV (n=102)	nOPV2 (n=300)	bOPV (n=300)
Sex							
	Female	659 (49·7%)	111 (47·0%)	338 (49·6%)	46 (45·1%)	154 (51·3%)	142 (47·3%)
	Male	666 (50·3%)	125 (53·0%)	344 (50·4%)	56 (54·9%)	146 (48·7%)	158 (52·7%)
Race							
	Black African	1322 (99·8%)	236 (100%)	682 (100%)	102 (100%)	300 (100%)	300 (100%)
	Other	3 (0·2%)	..	..	..	..	..
Ethnicity							
	Mandinka	750 (56·6%)	135 (57·2%)	389 (57·0%)	57 (55·9%)	166 (55·3%)	179 (59·7%)
	Wolof	107 (8·1%)	18 (7·6%)	48 (7·0%)	7 (6·9%)	23 (7·7%)	20 (6·7%)
	Fula	147 (11·1%)	31 (13·1%)	80 (11·7%)	13 (12·7%)	40 (13·3%)	25 (8·3%)
	Jola	154 (11·6%)	22 (9·3%)	73 (10·7%)	10 (9·8%)	36 (12·0%)	39 (13·0%)
	Other	167 (12·6%)	30 (12·7%)	92 (13·5%)	15 (14·7%)	35 (11·7%)	37 (12·3%)
Weight for height Z score							
	≥2 SD	1202 (90·7%)	222 (94·1%)	626 (91·8%)	93 (91·2%)	281 (93·7%)	285 (95·0%)
	<–2 SD*	123 (9·3%)	14 (5·9%)	56 (8·2%)	9 (8·8%)	19 (6·3%)	15 (5·0%)
Median age at screening (minimum to maximum value)		33 weeks (18 to 52)	30 weeks (21 to 52)	32 weeks (20 to 51)	34 months (19 to 52)	19 months (12 to 51)	19 months (12 to 56)

Data are n (%) unless otherwise stated. nOPV2=novel oral poliovirus vaccine type 2. bOPV=bivalent oral poliovirus vaccine.

* Indicating at least moderate malnutrition.

On the basis of post-dose one infant type 2 SNA seroconversion rates in the per-protocol population —the primary immunogenicity outcome—the pre-specified equivalence criteria for each of the three pairwise lot-to-lot comparisons were met (table 2; appendix p 23). Seroconversion rates across lots ranged from 315 (48·9%) of 644 (95% CI 45·0–52·9) to 318 (49·2%) of 646 (45·3–53·2). Point estimates for the between-lot differences in seroconversion rates ranged from –0·1% to –0·3%. The minimum lower bound of the 95% CIs for the difference in seroconversion rates was –5·8%. The maximum upper bound was 5·4%. The distribution of type 2 SNA antibodies across the three lots at the same timepoint was similar (appendix p 26). Supplementary comparisons of seroprotection rates and geometric mean SNA titre also indicated consistent immune responses across lots (appendix p 25). There was no change in inference when equivalence was assessed in the full analysis population. All further analysis in infants combines data for the three nOPV2 manufacturing lots.

**Table 2:** Poliovirus type 2 seroconversion primary lot-to-lot equivalence analysis—one dose infant per-protocol population

	**nOPV2**			Pairwise comparisons*		
	Lot 1	Lot 2	Lot 3	Lot 1 *vs* lot 2	Lot 1 *vs* lot 3	Lot 2 *vs* lot 3
n/N (%)	315/644 (48·9%)	318/649 (49·0%)	318/646 (49·2%)	−0·1	−0·3	−0·2
Exact 95% CI†	45·0 to 52·9	45·1 to 52·9	45·3 to 53·2	−5·5 to 5·4	−5·8 to 5·1	−5·7 to 5·2

Seroconversion rate is defined as either a post-dose type 2 reciprocal neutralising antibody titre of >8 in infants who were seronegative at baseline (type 2 reciprocal neutralising antibody titre of <8) or a four-fold rise in post-dose type 2 reciprocal neutralising antibody titres in infants who were seropositive at baseline including only those infants for whom a four-fold rise from baseline was possible based on the upper limit of quantification of the assay. Two-sided Miettinen and Nurminen 95% CI around differences in seroconversion rates. nOPV2=novel oral poliovirus vaccine type 2.

* Lot-to-lot equivalence was shown based on seroconversion rates if the 95% CIs around the difference between all pairwise lot-to-lot comparisons were contained within the −10% to 10% equivalence interval.

† Exact Clopper Pearson 95% CIs around seroconversion rates.

**Figure 2:**
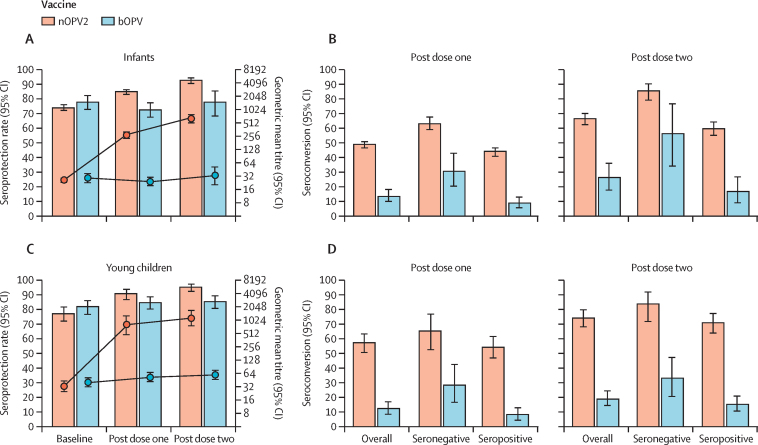
Poliovirus type 2 serum neutralising antibody seroprotection rates, geometric mean titres, and seroconversion rates in infants and young children (A) Infant seroprotection rates and geometric mean poliovirus type 2 neutralising antibody concentrations. (B) Infant post-dose one and post-dose two overall seroconversion rates and seroconversion rates in baseline seronegative and baseline seropositive infants. (C) Young children seroprotection rates and geometric mean poliovirus type 2 neutralising antibody concentrations. (D) Young children post-dose one and post-dose two overall seroconversion rates and seroconversion rates in baseline seronegative and baseline seropositive young children. Solid bars indicate seroprotection or seroconversion rates (left axis). Lines represent geometric mean type 2 serum neutralising antibody titres (right axis). Geometric mean titres and 95% CIs are estimated via the maximum likelihood based on survival methods including censoring at the lower limit of quantification and upper limit of quantification when appropriate. Seroprotection is defined as the percentage of participants with a reciprocal serum neutralising antibody titre of 8 or higher. Seroconversion (baseline seronegative) is defined as the percentage of participants with a post-dose reciprocal serum neutralising antibody titre of 8 or higher among those participants with a baseline reciprocal serum neutralising antibody titre of less than 8. Seroconversion (baseline seropositive) is defined as the percentage of participants with a post-dose four-fold rise in reciprocal serum neutralising antibody titres among those with a baseline reciprocal serum neutralising antibody titre of 8 or higher. This includes only those participants in whom a four-fold rise in reciprocal neutralising antibody titres was evaluable based on the baseline titre and upper limit of quantification for the assay. Seroconversion overall combined the seronegative and seropositive participants undergoing seroconversion. Exact Clopper-Pearson CIs are provided for seroprotection and seroconversion rates.

The seroprotection rate at baseline in infants in the nOPV2 groups was 1447 (74·4%) of 1949 (95% CI 72·4–76·3) increasing to 1656 (85·1%; 83·4–86·7) post-dose one and 604 (92·9%) of 650 (90·7–94·8) post-dose two (table 3, figure 2). The one dose seroconversion rate in infants who were seronegative at baseline was 316 (63·3%) of 499 (95% CI 58·9–67·6) increasing to 143 (85·6%) of 167 (79·4–90·6) after two doses (figure 2). In infants who were seropositive at baseline, the one-dose seroconversion rate was 635 (44·1%) of 1440 (95% CI 41·5–46·7) and the two dose was 288 (60·1%) of 479 (55·6–64·5). There was a substantial increase in geometric mean SNA titres in infants from a baseline of 26·7 (95% CI 23·9–29·7) to 277·9 (237·5–325·3) post-dose one (geometric mean fold rise 10·2; 95% CI 8·8–11·9) and 654·9 (524·5–817·7) post-dose two (24·1; 18·8–30·9). Overall, 25 (26·3%) of 95 (95% CI 17·8–36·4) infants in the bOPV group seroconverted to poliovirus type 2 over the course of two bOPV doses although there was no notable change in geometric mean antibody titres.

**Table 3:** Baseline, post-dose one, and post-dose two type 2 poliovirus serum neutralising antibody responses in infants and young children

		**Infants**		**Young children**	
		nOPV2 (n=1946)	bOPV (n=326)	nOPV2 (n=292)	bOPV (n=295)
**Baseline**					
Median (95% CI)*		4·17 (4·17–4·50)	4·50 (4·17–4·83)	4·83 (4·50–5·17)	5·17 (4·83–5·50)
Geometric mean titre (95% CI)†		26·7 (23·9–29·7)	29·3 (23·0–37·3)	33·2 (25·0–44·1)	41·3 (32·3–52·8)
n; seroprotection, % (95% CI)		1447; 74·4% (72·4–76·3)	255; 78·2% (73·3–82·6)	226; 77·4% (72·2–82·1)	242; 82·0% (77·2–86·2)
**Post-dose one**					
Median (95% CI)*		8·17 (7·50–8·83)	4·17 (3·83–4·50)	10·17 (8·83–10·34)	5·50 (5·17–5·83)
Geometric mean titre (95% CI)†		277·9 (237·5–325·3)	24·8 (19·6–31·4)	804·8 (490·4–1320·7)	53·4 (41·8–68·2)
n; seroprotection, % (95% CI)		1656; 85·1% (83·4–86·7)	238; 73·0% (67·8–77·8)	265; 90·8% (86·8–93·8)	250; 84·7% (80·1–88·7)
Seroconversion from baseline					
	n/N; baseline seronegative, % (95% CI)	316/499; 63·3% (58·9–67·6)	22/71; 31·0% (20·5–43·1)	43/66; 65·2% (52·4–76·5)	15/53; 28·3% (16·8–42·4)
	n/N; baseline seropositive, % (95% CI)	635/1440; 44·1% (41·5–46·7)	23/254; 9·1% (5·8–13·3)	100/185; 54·1% (46·6–61·4)	17/203; 8·4% (5·0–13·1)
	n/N; overall, % (95% CI)	951/1939; 49·0% (46·8–51·3)	45/325; 13·8% (10·3–18·1)	143/251; 57·0% (50·6–63·2)	32/256; 12·5% (8·7–17·2)
Post-dose one *vs* baseline geometric mean fold rise (95% CI)		10·2 (8·8–11·9)	0·9 (0·7–1·0)	7·4 (5·6–9·7)	1·2‡ (1·1–1·4)
**Post-dose two**					
Median (95% CI)*		10·17 (9·83–10·83)	4·50 (4·17–5·50)	10·17 (9·83–≥10·5)	5·83 (5·50–6·17)
Geometric mean titre (95% CI)†		654·9 (524·5–817·7)	33·5 (20·7–54·1)	1151·8 (774·6–1712·6)	60·2 (46·5–77·9)
n; seroprotection, % (95% CI)		604; 92·9% (90·7–94·8)	75; 78·1% (68·5–85·9)	276; 95·5% (92·4–97·6)	249; 85·6% (81·0–89·4)
Seroconversion from baseline					
	n/N; baseline seronegative, % (95% CI)	143/167; 85·6% (79·4–90·6)	13/23; 56·5% (34·5–76·8)	54/65; 83·1% (71·7–91·2)	17/52; 32·7% (20·3–47·1)
	n/N; baseline seropositive, % (95% CI)	288/479; 60·1% (55·6–64·5)	12/72; 16·7% (8·9–27·3)	129/183; 70·5% (63·3–77·0)	30/200; 15·0% (10·4–20·7)
	n/N; overall, % (95% CI)	431/646; 66·7% (62·9–70·4)	25/95; 26·3% (17·8–36·4)	183/248; 73·8% (67·9–79·2)	47/252; 18·7% (14·0–24·0)
N; post-dose two *vs* baseline geometric mean fold rise (95% CI)		658; 24·1 (18·8–30·9)	96; 1·0 (0·7–1·4)	289; 12·0 (9·2–15·6)	295; 1·4‡ (1·2–1·6)
Seroconversion from post-dose one					
	n/N; baseline seronegative, % (95% CI)	48/167; 28·7% (22·0–36·2)	7/23; 30·4% (13·2–52·9)	15/32; 46·9% (29·1–65·3)	10/51; 19·6% (9·8–33·1)
	n/N; baseline seropositive, % (95% CI)	117/471; 24·8% (21·0–29·0)	9/73; 12·3% (5·8–22·1)	35/92; 38·0% (28·1–48·8)	22/192; 11·5% (7·3–16·8)
	n/N; overall, % (95% CI)	165/638; 25·9% (22·5–29·4)	16/96; 16·7% (9·8–25·7)	50/124; 40·3% (31·6–49·5)	32/243; 13·2% (9·2–18·1)
N; post-dose two *vs* post-dose one geometric mean fold rise (95% CI)		658; 2·0 (1·6–2·5)	96; 1·1 (0·9–1·5)	289; 1·6 (1·3–1·9)	295; 1·1 (1·0–1·3)

Seroprotection is defined as the percentage of participants with a reciprocal serum neutralising antibody titre of >8. Seroconversion (baseline seronegative) is defined as the percentage of participants with a post-dose reciprocal serum neutralising antibody titre of ≥8 among those participants with a baseline reciprocal serum neutralising antibody titre of <8. Seroconversion (baseline seropositive) is defined as the percentage of participants with a post-dose four-fold rise in reciprocal serum neutralising antibody titres among those with a baseline reciprocal serum neutralising antibody titre of ≥8. This includes only those participants in whom a four-fold rise in reciprocal neutralising antibody titre was evaluable based on the baseline titre and upper limit of quantification for the assay. Seroconversion overall combined the seronegative and seropositive participants undergoing seroconversion. When only participants with a result within the limits of quantification were included the post-dose 1 type 2 serum neutralising antibodies geometric mean fold rise following bOPV was 1·1 (95% CI 1·02–1·29, n=216) and the post-dose two type 2 serum neutralising antibodies geometric mean fold rise following bOPV was 1·1 (0·95–1·20, n=205). Exact Clopper-Pearson CIs are provided around seroprotection and seroconversion rates. Geometric mean fold rise and 95% CIs are unadjusted asymptotic geometric mean fold rise parameter estimates. bOPV=bivalent oral poliovirus vaccine. nOPV2=novel type 2 oral poliovirus vaccine.

* 95% CIs are estimated via the percentile bootstrap method (10 000 simulations).

† Geometric mean titre and 95% CIs are maximum likelihood parameters estimated based on survival methods including censoring at the lower limit of quantification (LLOQ) and upper limit of quantification when appropriate.

‡ Due to the number of young children below the LLOQ with both the baseline and post-baseline value, in whom the geometric mean fold rise was calculated as LLOQ ÷ [LLOQ ÷ 2] = 2, the geometric mean fold rise is falsely inflated.

The seroprotection rate at baseline in young children in the nOPV2 groups was 226 (77·4%) of 292 (95% CI 72·2–82·1) increasing to 265 (90·8%; 86·8–93·8) post-dose one, and 276 (95·5%) of 289 (92·4–97·6) post-dose two (table 3, figure 2). The one-dose seroconversion rate in young children who were seronegative at baseline was 43 (65·2%) of 66 (95% CI 52·4–76·5), increasing to 54 (83·1%) of 65 (71·7–91·2) after two doses (figure 2). In young children who were seropositive at baseline, the one-dose seroconversion rate was 100 (54·1%) of 185 (95% CI 46·6–61·4) and the two-dose was 129 (70·5%) of 183 (95% CI 63·3–77·0). There was a substantial increase in geometric mean SNA titres in young children from a baseline of 33·2 (95% CI 25·0–44·1) to 804·8 (95% CI 490·4–1320·7) post-dose one (geometric mean fold rise 7·4; 95% CI 5·6–9·7) and 1151·8 (774·6–1712·6) post-dose two (12·0; 9·2–15·6]). Overall, 47 (18·7%) of 252 (95% CI 14·0–24·0) young children in the bOPV group seroconverted to poliovirus type 2 over the course of two bOPV doses. The distributions of type 2 SNA antibodies at each timepoint are illustrated in the appendix (p 26).

273 (40%) of 682 (95% CI 36–44) infants in the reactogenicity population experienced at least one solicited adverse event following the first or second dose of nOPV2 compared with 47 (46%) of 102 (36–56) infants following the first or second dose of bOPV (table 4). There was one severe solicited fever (>39·1°C) in an infant who received bOPV. There were no severe solicited events in infants who received nOPV2 (appendix p 27). Among solicited events, fever of 37·5°C or higher was more common in infants following bOPV (26 [25·5%] of 102; 95% CI 17·4–35·1) than following nOPV2 (105 [15·4%] of 682; 12·8–18·3; p=0·015). There were no other notable differences in the rates of solicited adverse events.

**Table 4:** Solicited and unsolicited safety events in infants and young children

	**Infants receiving nOPV2**		**Infants receiving bOPV**			**Young children receiving nOPV2**		**Young children receiving bOPV**	
	Number of events*	n/N (%) 95% CI	Number of events*	n/N (%) 95% CI	p value	Number of events*	n/N (%) 95% CI	Number of events*	n/N (%) 95% CI
**Any solicited adverse event within 7 days of vaccination**†									
Any severity	456	273/682‡ (40%) 36–44	80	47/102‡ (46%) 36–56	..	149	98/300‡ (33%) 27–38	153	98/300‡ (33%) 27–38
Severe	0	0/682‡ (0%) 0–1	1	1/102‡ (1%) 0–5	..	4	4/300‡ (1%) <1–3	2	2/300‡ (<1%) <1–2
**Unsolicited adverse event to 28 days of last vaccination**									
Any severity	1094	837/2007§ (42%) 40–44	197	161/338§ (48%) 42–53	p=0·043¶	248	166/300§ (55%) 50–61	248	172/300§ (57%) 52–63
Severe	4	4/2007§ (<1%) 0–1	2	2/338§ (<1%) 0–2	..	0	0/300§ (0%) 0–1	1	1/300§ (<1%)0–1
**Related unsolicited adverse event to 28 days of last dose**									
Any severity	14	14/2007§ (<1%) 0–1	3	3/338§ (<1%) 0–3	..	0	0/300§ (0%) 0–1	0	0/300§ (0%) 0–1
Severe	0	0/2007§ (0%) 0–0	0	0/338§ (0%) 0–1	..	0	0/300§ (0%) 0–1	0	0/300§ (0%) 0–1
Serious adverse events	18	17/2007§ (<1%) <1–1	5	5/338§ (2%) 1–3	..	1	1/300§ (0%) 0–2	4	4/300§ (1%) 0–3
Related serious adverse events	0	0/2007§ (0%) 0–0	0	0/338§ (0%)0–1	..	0	0/300§ (0%) 0–1	1	1/300§ (0%) 0–2

95% CIs calculated using the exact Clopper-Pearson method. bOPV=bivalent oral poliovirus vaccine. nOPV2=novel type 2 oral poliovirus vaccine.

* Number of participants included in the analysis of the given population (reactogenicity or safety).

† Fever (axillary temperature >37·5°C), vomiting, diarrhoea, irritability, decreased feeding, and decreased activity (including immediate solicited events occurring at 30 min post-dose in the reactogenicity population).

‡ Reactogenicity population.

§ Safety population.

¶ Fisher's exact two-tailed test of the rate of events between nOPV2 and bOPV. No other difference in event rates between nOPV2 and bOPV within the age cohorts was significant at the 5% significance level.

98 (33%) of 300 (95% CI 27–38) young children had a solicited adverse event following a first or second dose of both nOPV2 and bOPV (table 4). There were four severe solicited events, all of which were fevers of 39·1°C or higher, in young children who received nOPV2 (appendix p 27). All the children recovered with no more than simple antipyretic medications. Two young children had a severe solicited adverse event following bOPV. The first, a fever of 39·1°C or higher, resolved with antipyretic medication while the second with severe vomiting, required hospitalisation and was thus recorded as a solicited serious adverse event.

837 (42%) of 2007 (95% CI 40–44) infants experienced an unsolicited adverse event within 28 days of nOPV2 administration compared with 161 (48%) of 338 (42–53) infants in the same time interval following bOPV (p=0·043; table 4). Four of these events (<1%; 95% CI 0–1) were severe in the nOPV2 group, whereas two (<1%; 0–2) were severe in the bOPV group. None of the severe events were judged to be related to vaccination. Upper respiratory tract infections and gastroenteritis were the most common unsolicited adverse events in infants (appendix p 28). There were no clinically meaningful differences in the frequencies of any common unsolicited events between groups. 14 infants (<1%; 95% CI 0–1) had an unsolicited adverse event judged to be related to vaccination following nOPV2. Three infants (<1%; 95% CI 0–3) had an unsolicited adverse event judged to be related to vaccination following bOPV. The majority of these events occurred in the first week after vaccination in infants who were not in the reactogenicity group.

Of the young children who received nOPV2, 166 (55%) of 300 (95% CI 50–61) experienced an unsolicited adverse event within 28 days of vaccination compared with 172 (57%) of 300 (52–63) young children who received bOPV (table 4). There were no severe unsolicited adverse events in the same time period following nOPV2 and one severe event following bOPV. Upper respiratory tract infections and gastroenteritis were again the most common unsolicited adverse events in young children and occurred at a similar frequency in the two vaccine groups (appendix p 29). There were no unsolicited adverse events judged to be related to vaccination in young children.

There were 18 serious adverse events in 17 infants (<1%; 95% CI <1–1·4) following nOPV2 and five events in five infants (2%; <1–3) following bOPV (table 4; appendix pp 30–31). These events included one death in a house fire in an infant in the nOPV2 group and one death secondary to sepsis in the bOPV group. None of the serious adverse events in infants were considered to be related to vaccination. In young children, there were one serious adverse event following nOPV2 and four in four children following bOPV. Other than the solicited vomiting requiring hospital admission on day three after receipt of bOPV, none of the other serious adverse events in young children were considered to be related to vaccination.

Among the 293 infants contributing a pre-vaccination stool sample, 5 (2%) were positive for type 2 poliovirus (appendix p 32). Among the viral shedding population, which was limited to only those who were type 2 negative before vaccination, 7 days following nOPV2, 78 (42%) of 187 (95% CI 35–49) were excreting a type 2 virus. By 28 days following vaccination this decreased to 8 (4%) of 186 (95% CI 2–8). No type 2 poliovirus was detected 84 days following vaccination. The estimated median time to cessation of viral shedding was 7 days. Following bOPV, 7 (21%) of 34 (95% CI 9–38) of those tested were excreting a type 1 poliovirus and 9 (27%; 13–44) were excreting a type 3 poliovirus 7 days after vaccination. None of the samples from infants who received bOPV tested positive for poliovirus 28 days following vaccination. A single bOPV recipient (3%) tested positive for type 2 virus in stool at day 8.

## Discussion

This phase 3 trial provides key safety and immunogenicity data required for the licensure and WHO prequalification of nOPV2. Lot-to-lot equivalence was shown. The vaccine was safe, based on the first contemporaneous double-blind comparison with bOPV. A rapid decrease in faecal shedding of the virus in infants, who had not previously been primed enterally with a type 2 polio vaccine, was shown.

The two-dose seroconversion rates of between two-thirds and three-quarters, in infants and young children in this trial, were lower than those reported in the phase 2 trial of nOPV2 in Panama in which close to 100% of infants and children seroconverted.^10^ However, among those infants and young children who were seronegative at baseline, more than 80% seroconverted following two nOPV2 doses. This resulted in a post-dose two seroprotection rate of around 95% in both age groups, which is reassuring in the context of the future use of nOPV2 in cVDPV2 outbreak response campaigns.

The lower seroconversion rates and post-dose shedding rates in this study in The Gambia, compared with the rates in Panama do not appear to be explained by differences in previous polio vaccination history, which were similar between the two studies. They are instead consistent with findings for mOPV2 and other enteric vaccines in low-income countries.^20^ Gut inflammation, associated with environmental enteropathy as well as co-infection with other non-polio enteroviruses are thought to go some way to explain this.^20^ A review summarising the results of studies with mOPV2 conducted between the 1950s and 1980s, showed single dose type 2 seroconversion rates of between 77% and 100% across different populations.^21^ Studies conducted in temperate regions tended to show higher seroconversion rates than those conducted in non-temperate regions such at The Gambia. A trial conducted in inactivated poliovirus vaccine vaccinated infants aged 11–18 months in Mozambique showed a type 2 seroconversion rate of 83 (60·6%) of 137 (95% CI 52·2–68·4) following two mOPV2 doses.^22^ This finding contrasts with a seroconversion rate of 88 (92%) of 96 (95% CI 84–96) in historic control infants following one dose of mOPV2 in the study in Panama.^10^ Thus, the immunogenicity of nOPV2 in this trial is consistent with data for mOPV2 from comparable settings in sub-Saharan Africa.

The seroprotection rates of more than 90% in both infants and young children following two nOPV2 doses contrasts with data from Liberia, collected in the context of an outbreak response campaign, where the post-campaign type 2 seroprevalence was 144 (42·1%) of 342 (95% CI 36·8–47·5) in children who had received two nOPV2 doses based on parental recall.^23^ Given the close geographical location of The Gambia and Liberia and their comparable socioeconomic statuses, the trial results are reassuring as they exclude profoundly lower vaccine immunogenicity in non-temperate west Africa as the primary driver of the low seroprotection levels reported from Liberia.^24^, ^25^ An absence of previous inactivated poliovirus vaccine immunisation in a proportion of participants in Liberia, technical issues related to the Liberia campaign or study, and inaccurate coverage data based on parental recall, are alternative explanations.^26^ Such issues were avoided in the context of this rigorously controlled phase 3 trial but should be accounted for in public health policy decisions.

The lot-to-lot equivalence data are reassuring in showing that the genetic heterogeneity in lots, generated during routine production, does not affect immunogenicity.^27^ In isolation, the VP1-E295K variant, present at levels between 25% and 44% across the three lots used in this trial, reduces nOPV2 viral fitness and immunogenicity in a transgenic mouse model.^27^ However, it's non-random co-location on genomes, with VP1-N171D, which reduced attenuation, has been shown to phenotypically offset this defect and is consistent with the lot-to-lot equivalence shown. Non-random co-location of the two mutations in shed virus from nOPV2 recipients in the trials in Panama further support this finding.^14^ The co-location similarly offsets the reduction in attenuation associated with VP1-N171D.^27^

Modelling based on cVDPV2 isolates and nOPV2 and mOPV2 delivered through supplementary immunisation activities conducted across Nigeria between 2016 and 2022 estimated the vaccines reduced the susceptible population by 42% (95% CI 28 to 54) and 38% (20 to 51) respectively.^28^ A retrospective case–control study from the same setting recently reported a one-dose effectiveness against acute flaccid paralysis of 12% (95% CI –2 to 25) for nOPV2 and 17% (3 to 29) for mOPV2.^29^ These data support the comparability of the responses to mOPV2 and nOPV2. However, they also highlight the disconnect between seroprotection rates reported in trials and field effectiveness even in close geographically and socioeconomically related countries. Thus, increases in the number of high-quality supplementary immunisation activities are crucial if sufficient population immunity to avert outbreaks is to be generated.^2^, ^30^, ^31^

More than a quarter of infants and close to one-fifth of young children seroconverted to poliovirus type 2 following bOPV. Heterotypic immune responses, to serotypes not contained in the vaccine, are well described and typically transient.^32^ One (3%) of 34 infants was positive for the type 2 virus in their stool on day 7 following bOPV. This could reflect exposure to either environmental cVDPV2 or to nOPV2 from other trial participants but indicates accidental administration of the incorrect vaccine is unlikely to have contributed to the type 2 seroconversion in this group.

The trial had several strengths. It provides the first trial data on nOPV2 from sub-Saharan Africa—the region that has experienced the greatest burden of cVDPV2 outbreaks since the switch from trivalent OPV to bOPV. Thus, the data are of direct relevance to a key target population for nOPV2 use going forward. Although VDPV2 was detected in environmental samples in The Gambia around the time of the trial and in a similar geographical location, a small fraction of infants were excreting a type 2 poliovirus at the time of enrolment. Therefore, although a very low level of enteral priming through previous exposure to VDPV2 in participants cannot be excluded, the serological responses generated are representative of those of the key target population who have not been primed against poliovirus type 2 by the mucosal route.

The trial had several limitations. First, the absence of an active control vaccine for the type 2 immunogenicity and viral excretion data was unavoidable given the restrictions on the usage of the type 2 Sabin OPV. Second, despite the comparison of safety data with bOPV and the substantial number of infants and children vaccinated in this study that when combined with data from the phase 2 trials provides reassurance regarding the safety profile of nOPV2, rare safety events might not have been detected and safety surveillance during deployment under the Emergency Use Listing and subsequently following licensure remains important.

In summary, this trial reports the crucial data required to support the licensure and WHO prequalification of nOPV2. When delivered through high-quality cVDPV2 outbreak response campaigns, the vaccine is expected to effectively interrupt viral transmission while being associated with a considerably reduced risk of seeding further paralytic outbreaks, even in OPV2-naive populations, compared with Sabin OPV2. The vaccine is expected to be a vital tool in the polio eradication endgame strategy.^2^

## Data sharing

The individual participant data that underlie the results reported in this Article, after de-identification (ie, text, tables, figures, and appendices), will be shared. They will be available beginning 3 months and ending 3 years after publication. Supporting clinical documents including the study protocol, statistical analysis plan, and the informed consent form will be available immediately following publication on application to the corresponding author. Researchers who provide a scientifically sound proposal will be allowed access to the individual participant data. Proposals should be directed to the corresponding author. These proposals will be reviewed and approved by the funder, investigator, and collaborators based on scientific merit. To gain access, data requesters will need to sign a data access agreement.

## Declaration of interests
