# Supplementary material for: Safety of the novel oral poliovirus vaccine type 2 (nOPV2) in infants and young children aged 1 to <5 years and lot-to-lot consistency of the immune response to nOPV2 in infants in The Gambia: a phase 3, double-blind, randomised controlled trial
Source: Lancet. 2024 Mar 23;403(10432):1164–75. doi: 10.1016/S0140-6736(23)02844-1 (PMC10985839; doi:10.1016/S0140-6736(23)02844-1)
Supplement: Supplementary appendix [file mmc1.pdf]

THE LANCET

Supplementary appendix

This appendix formed part of the original submission and has been peer reviewed. We post it as supplied by the authors.

Supplement to: Ochoge M, Futa AC, Umesi A, et al. Safety of the novel oral poliovirus vaccine type 2 (nOPV2) in infants and young children aged 1 to <5 years and lot-to-lot consistency of the immune response to nOPV2 in infants in The Gambia: a phase 3, double-blind, randomised controlled trial. *Lancet* 2024; published online Feb 22. https://doi.org/10.1016/S0140-6736(23)02844-1.

Contents

Appendix: Inclusion and Exclusion Criteria 2
Inclusion criteria 2
Exclusion criteria 3
Supplementary figure: Study schedule 6
Supplementary table: Genetic heterogeneity of lots used for the lot-to-lot consistency. 7
Appendix: US Centers for Disease Control and Prevention. 8
Supplementary table: grading of solicited adverse events. 9
Supplementary table: functional grading of unsolicited adverse events. 10
Appendix: Sample size considerations 11
Primary immunogenicity evaluation 11
Primary safety evaluation 12
Infants 12
Young children 15
Appendix: Analysis populations 20
Supplementary table: baseline demographic and anthropometric characteristics in infants enrolled in the safety population according to lot received 21
Supplementary table: prior polio vaccination history in infants and young children – safety population. 22
Supplementary figure: poliovirus type 2 lot to lot equivalence figure – one dose infant per protocol population 23
Supplementary table: poliovirus type 2 lot-to-lot seroprotection and geometric mean titre equivalence – one dose infant per protocol population. 25
Supplementary figure: poliovirus type 2 serum neutralizing antibody reverse cumulative distribution curves in infants and children at the indicated timepoints. 26
Supplementary table: solicited adverse events collected between the day of vaccination and day seven after vaccination in the infant and young children reactogenicity cohorts. 27
Supplementary table: unsolicited adverse events occurring in ≥2% of infants. 28
Supplementary table: unsolicited adverse events occurring in ≥2% of young children. 29
Supplementary table: serious adverse event listing – infants and young children 30
Supplementary table: poliovirus shedding rates among infants. 32
Supplementary table: cessation of poliovirus type 2 viral shedding following a single nOPV2 dose. . 32

Appendix: Inclusion and Exclusion Criteria

To be eligible for enrolment (study vaccination), participants must have met all the inclusion criteria and none of the exclusion criteria for the study. The investigator should always use good clinical judgement in considering a participant's overall eligibility based on the inclusion and exclusion criteria.

Inclusion criteria

1. Age:
   - Infants: ≥18 and <52 weeks at the time of first study vaccination (126 days through the day before their first birthday, inclusive, with the day after birth considered 1-day old)
   - Young children: ≥1 to <5 years old at the time of the first study vaccination (from the first birthday up to the day prior to the fifth birthday)
     - Should an OPV2 vaccine campaign occur during the study, the upper age limit of the young child cohort with be expanded to <7 years old (up to the day prior to the seventh birthday).
2. The participant's parent must be judged to be willing and able to provide informed consent based on the content of the informed consent document and signed/thumb-printed informed consent must be provided.
3. Intention of the participants' parents to remain in the study area with the infant/young child during the study period.
4. The participant must have a readily identifiable place of residence in the study area.
5. Infants: prior receipt of full primary series of bOPV and a single dose of IPV prior to randomization, with IPV dose at least 4 weeks prior to randomization
6. Young children: prior receipt of at least one dose of type 2-containing vaccine (IPV, tOPV or mOPV2), with the last dose of type 2-containing vaccine at least 4 weeks prior to the day of first study vaccination.

   [The polio vaccination histories required for eligibility in infants and young children reflect the minimum expected routine protection against poliovirus type 2 in children of the respective age

groups in The Gambia schedule which also aligns with WHO recommendations. The requirements aims to ensure infants and young children are not potentially disadvantaged with regards to their polio type 2 protection through trial enrolment. Young children who have not received type 2 containing vaccine may receive IPV and become eligible to be randomized to receive study vaccine 4 weeks after receipt of IPV.]

7. Parent ability and willingness to comply with the required study procedures, including the home visits, clinic visits, assessment and sampling procedures, as judged by the investigator.
8. Parent willingness to contact the study team in the event of an acute illness.
9. Willingness not to use herbal and other traditional medications for the duration of the study.
10. Infant Welfare Card (IWC) is available.
11. Baseline peripheral blood sample obtained from the infant/young child that is sufficient to allow the primary trial immunogenicity endpoints to be evaluated.

Exclusion criteria

1. Moderate or severe (grade ≥ 2) acute illness at the time of enrollment/first study vaccination – temporary exclusion (see Appendix 2: Severity Grading Tables)
   - Participant with mild (grade 1) acute illnesses may be enrolled at the discretion of the investigator.
2. Presence of fever on the day of enrollment/first study vaccination (axillary temperature ≥37.5ºC) – temporary exclusion.
3. Presence of abnormal vital signs (respiratory rate and/or heart rate) for age on the day of enrollment/first study vaccination – temporary exclusion.
4. Receipt of any investigational medicinal product within six months of study enrollment or intended. receipt of any investigational medicinal product at any time during study participation.
5. Concurrent participation or intent to participate in another clinical trial or other study throughout the entire timeframe for this study.

6. Presence of severe malnutrition [weight-for- length/height z-score <-3SD median (per WHO published child growth standards)] – temporary exclusion if marginal and subsequently gains weight.
7. Presence of any clinically significant systemic disorder (cardiovascular, respiratory, hepatic, renal, gastrointestinal, hematological, endocrine, dermatological, neurological, cancer or autoimmune disease) as determined by medical history and/or physical examination that could compromise the participant's health, is likely to result in nonconformance to the protocol or is likely to interfere with the evaluation of safety or immunogenicity endpoints.
8. History or examination findings suggestive of a primary or secondary immunodeficiency or known maternal HIV infection.
9. Household member (living under the same roof/in the same building rather than in the same compound) with a history indicating or suggestive of a primary or secondary immunodeficiency.
10. Evidence of a clinically significant congenital or genetic defect as judged by the investigator.
11. Known sensitivity or allergy to any components of the study vaccine.
12. History of anaphylactic reaction.
13. Infant or young child of a mother of an infant or young child already enrolled in the trial.
14. Receipt of any vaccine within 28 days of day of study vaccination or intent to administer non-study vaccine prior to the post-vaccination blood sample.
15. Receipt of any immunoglobulin therapy and/or blood products in the past 6 months or planned administration during the study.
16. History of chronic administration (defined as more than 14 days) of immunosuppressant medications, including corticosteroids ($\geq$ 0.5mg/kg/day of prednisolone (or equivalent). Topical and inhaler steroids are permitted (unless indicative of a significant chronic illness otherwise excluding the infant/young child).

17. The participant is a direct descendent (child or grandchild) of a person employed by the investigator site (MRCG at LSHTM), the sponsor (PATH), the clinical research organization or the vaccine manufacturer (Bio Farma).

18. Any other medical or social condition or other concern in the participant and/or parent which, based on the judgment of the investigator, may interfere with the assessment of the study objectives, pose a potential risk to the participant, or prevent the participant completing the required study procedures.

19. Following a vaccine campaign, infants born less than 4 weeks after an older sibling has received a campaign vaccine will be excluded. If the date of the older sibling's vaccination is unknown, the date of the last day of the campaign will be used for that determination.

In the case of temporary exclusions, a participant may be rescreened and enrolled at a later timepoint and still be enrolled. Re-screening cannot take place within 48 hours of a recorded fever (axillary temperature ≥37.5°C).

Supplementary figure: Study schedule

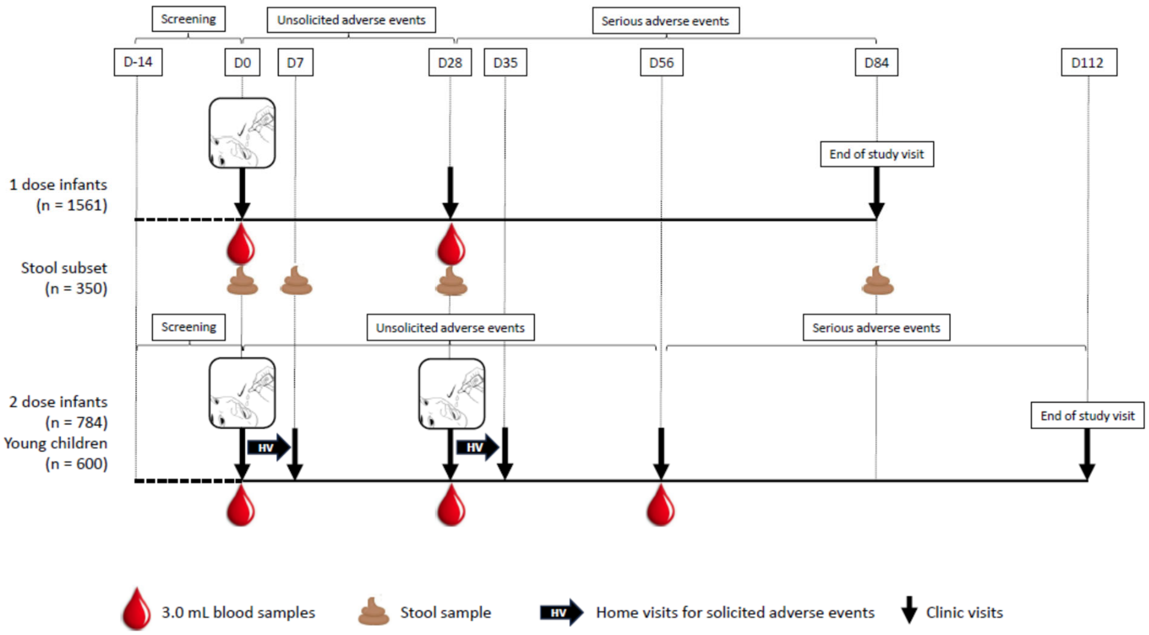

Supplementary table: Genetic heterogeneity of lots used for the lot-to-lot consistency.

Study Lot	Lot number	Potency, log $CCID_{50}$/ 0.1 mL	Standard NGS analysis % substitution			Colocation analysis, % reads with pair[1]			
			VP1-I143T	VP1-N171D	VP1-E295K	171N/ 295E	171D/ 295K	171D/ 295E	171N/ 295K
1	2220720	5.57	1.03	21.2	25.1	74	16	2	9
2	2220820	5.59	1.25	27.6	34.4	60	26	2	12
3	2220920	5.59	1.49	35.3	43.9	54	33	4	8

NGS – next generation sequencing; $CCID_{50}$ – cell culture infectious dose 50%; methodological details are provided.[1] Release specifications based on NGS data ensure that no mutations are detected in the modified regions of the virus and that amounts of selected mutations that result in amino acid substitutions are limited.[1] For the latter mutations, when assessed individually using molecular clones, two of these substitutions (I143T and N171D in viral protein 1 [VP1]) slightly reduce attenuation while one (E295K in VP1) decreases viral fitness at 37°C and reduces immunogenicity in mice; Colocation analysis focused on frequency of nucleotide substitutions at two positions: A3053G encoding for VP1-N171D substitution and G3425A encoding for VP1-E295K substitution. Percentage of each of the four combinations of parental (171N and 295E) and substituted (171D and 295K) amino acid is shown.

Interested readers are referred to reference 1 for a more detailed methodological introduction to this area.

Appendix: US Centers for Disease Control and Prevention.

This activity was reviewed by CDC, deemed not research, and was conducted consistent with applicable federal law and CDC policy (See e.g., 45 C.F.R. part 46, 21 C.F.R. part 56; 42 U.S.C. §241(d); 5 U.S.C. §552a; 44 U.S.C. §3501 et seq.).

Supplementary table: grading of solicited adverse events.

Reactogenicity	Mild (Grade 1)	Moderate (Grade 2)	Severe (Grade 3)	Potentially Life Threatening (Grade 4)
Acute systemic allergic reaction	Localized urticaria (wheals) with no medical intervention indicated	Localized urticaria with medical intervention indicated OR Mild angioedema with no medical intervention indicated	Generalized urticaria OR Angioedema with medical intervention indicated OR Symptomatic mild bronchospasm	Acute anaphylaxis OR Life-threatening bronchospasm OR laryngeal edema
Fever (axillary)	37.5 – 38.0°C	38.1 – 39.0°C	39.1 – 40.5°C	> 40.5°C
Vomiting	1 episode per 24 hours	2-5 episodes per 24 hours	≥6 episodes per 24 hours or requiring parenteral hydration	Life-threatening consequences (e.g., hypotensive shock)
Diarrhoea, <1 year of age	Liquid stools (more unformed than usual) but usual number of stools	Liquid stools with increased number of stools OR Mild dehydration	Liquid stools with moderate dehydration	Liquid stools resulting in severe dehydration with aggressive rehydration indicated OR Hypotensive shock
Diarrhoea, ≥1 year of age	Transient or intermittent episodes of unformed stools OR Increase of ≤ 3 stools over baseline per 24-hour period	Persistent episodes of unformed to watery stools OR Increase of 4 to 6 stools over baseline per 24-hour period	Increase of ≥ 7 stools per 24-hour period OR IV fluid replacement indicated	Life-threatening consequences (e.g., hypotensive shock)
Irritability	Crying more than usual but easily consoled	Crying more than usual and somewhat difficult to console	Continuous crying that is inconsolable	
Decreased activity	Slightly subdued, but responds normally to stimuli	Subdued and does not respond as readily as normal to stimuli	Lethargic	Obtunded
Decreased feeding	Eating less than normal	Missed 1 or 2 feeds/meals completely	Refuses ≥3 feeds/meals or refuses most feeds/meals	Life-threatening consequences OR Aggressive intervention indicated [e.g., tube feeding or total parenteral nutrition (TPN)]

Division of AIDS Tables for Grading the Severity of Adult and Pediatric Adverse Events (version 2.1, July 2017)

Supplementary table: functional grading of unsolicited adverse events.

Mild (Grade 1)	(Moderate (Grade 2)	Severe (Grade 3)	Potentially Life Threatening (Grade 4)
No or minimal interference with usual activities; no medical intervention/therapy required	Greater than minimal interference with usual activities; no or minimal medical intervention/therapy required	Marked limitation in ability to perform usual activities; medical intervention/therapy required.	ISNAility to perform basic functions OR Medical or operative intervention indicated to prevent permanent impairment, persistent disability, or death

Division of AIDS Tables for Grading the Severity of Adult and Pediatric Adverse Events (version 2.1, July 2017)

Appendix: Sample size considerations

Primary immunogenicity evaluation

The primary immunogenicity hypothesis to be tested is that each of three lots of the nOPV2 vaccine is equivalent to each other lot. This overall hypothesis will be evaluated by testing each pairwise comparison between lots and declaring joint equivalence if equivalence is established for each comparison independently. Equivalence of immunogenicity from each of the three lots will be evaluated through the seroconversion rate of serum neutralizing antibodies (SNA). Seroconversion is defined by minimum 4-fold increase in titre between baseline and 28 days post-vaccination among those initially seropositive, or seropositivity [reciprocal SNA titre ≥ 8] at 28 days among those initially seronegative. Based on a conservative estimate derived from preliminary results from the phase 2 study[2], it is anticipated that ≥4 weeks following the last bOPV/IPV doses, approximately 90% of participants receiving at least 3-doses of bOPV plus 1 IPV as a primary series will have SNA titres sufficiently below the assay upper limit of quantification (ULOQ) to enable observation of seroconversion ($\log_2$ titre ≤ 8.5). Among these participants, seroconversion is anticipated to be at least 80%. Equivalence between two lots will be assessed by computing the difference in seroconversion rate between the lots along with the two-sided 95% Miettinen and Nurminen confidence intervals [3,4]; equivalence will be declared if the confidence interval is contained entirely within the equivalence interval of (-10%, 10%). The equivalence interval was selected and agreed in advance with the WHO for the purposes of pre-qualification. It is judged sufficient to confirm consistency of manufacturing and has been widely used for the assessment of equivalence for other vaccines with comparable seroconversion rates. Lot-to-lot equivalence will be declared if the equivalence comparison is successful for all three pairwise comparisons. Additional assessments will compare the age distribution among lots as well as the baseline titre values and other variables anticipated to be related to immune response, with supplementary adjusted and/or subgroup comparisons performed if imbalance is detected.

Under a conservative assumption of independence of the between-lot comparisons. In order to have ≥90% power overall to declare manufacturing lot consistency, 536 participants per group must be available for evaluation of seroconversion at 28 days following the first dose. Power >90% is available for a seroconversion rate of >80%. Allowing for dropout due to high baseline titres as above, and

approximately 10% dropout for other reasons, 670 participants will be enrolled per group, for a total of 2010 participants for the lot-consistency study. In the event that dropout due to OPV2 vaccination campaigns prior to contribution of the primary endpoint sample is excessive, replacements may be enrolled to ensure adequate power for evaluation of the primary immunogenicity objective.

Primary safety evaluation

Infants

In recognition of the lack of a contemporaneous control in prior studies involving nOPV2, a bOPV control arm is included in the current study to provide reference data for the safety evaluation of the nOPV2 vaccine candidate. In order to provide sufficient and relevant safety reference data but also limit the size of the group receiving bOPV, the size of the bOPV group is obtained, after fixing the size of the groups receiving nOPV2 owing to the immunogenicity lot-to-lot consistency objective, as the size which permits a high probability of detecting an excess rate of unexpected adverse events in the nOPV2 arms compared to the bOPV control arm, following at least one dose. A sample size of 335 has been chosen to satisfy the above, as well to obtain a randomization ratio which can be easily implemented in practice.

Rates of adverse events by type, severity, and relationship will be compared using a two-sided level $\alpha = 0.05$ Fisher's exact test between the combined nOPV2 arms versus the bOPV control arm. The table below indicates the power, determined via simulation, to detect a difference between various assumed event rates in the two arms.

Table: Power to detect difference between various assumed event rates in two arms

Event Rate, Combined nOPV2 Groups (n=2010)	Event Rate, Control Group (n=335)	Difference (nOPV2 minus Control)	Power
1%	0%	1%	22%
2%	1%	1%	16%
2%	0%	2%	>99%
4%	1%	3%	90%
5%	1%	4%	98%
10%	5%	5%	89%
20%	5%	15%	>99%
20%	10%	10%	>99%
20%	17%	3%	24%
20%	15%	5%	58%
20%	12%	8%	96%

The minimum difference in event rate (surplus in the combined nOPV2 groups) that can be detected with ≥90% probability is 2% (at 2% in the combined nOPV2 groups and 0% in the bOPV control group), which is considered to be adequate. Among those scenarios with a non-zero event rate in the bOPV control group, the minimum difference detectable with ≥90% power is 3% (4% with nOPV2 and 1% with bOPV).

It is also of interest to have sufficient sample size to enable the detection of rare events with high probability. Given that 2010 participants are expected to receive a single dose of study vaccine and contribute at least some safety data, the table below displays the probability of detection of events (observation of 1 or more such events) with a specified true rate, in the combined nOPV2 arms. If 0, 1, or 2 events of a given type are detected among 2010 participants, the upper bounds of the 95% confidence interval are 0.18%, 0.28%, and 0.36%, respectively.

Table: Probability of detection of events specified true rate

Probability of observing at least one event	Minimum Event Rate Required
50%	0.04%
80%	0.08%
90%	0.12%
95%	0.15%
**Probability of observing at least two events**	**Minimum Event Rate Required**
50%	0.08%
80%	0.15%
90%	0.19%
95%	0.23%

Reactogenicity/post-dose-2 safety evaluation

For tolerability evaluation following 1 dose, and for safety and tolerability evaluation following a second dose, following randomization to nOPV2 or bOPV, participants in the nOPV2 arms will be further randomized 2:1 to receive 1 or 2 doses, with all participants receiving 2 doses contributing reactogenicity data during the 7-day period following each dose administration. All participants who are randomized to the bOPV control arm to receive 2 doses will also contribute reactogenicity data following both doses. Therefore, 672 nOPV2 participants and 112 bOPV participants will receive 2 doses.

The table below describes the power of the two-sided level $\alpha = 0.05$ Fisher's exact test, obtained via simulation, to detect an odds ratio (in the direction of higher rates for the nOPV2 arm only) of a given magnitude in the rate of participants experiencing any given solicited adverse events of a specified severity in the reactogenicity cohort. Power ≥ 89% is available for odds ratios ≥ 2.0 when the control rate is ≥10%, and power ≥97% is available for odds ratios ≥2.5 when the control rate is ≥7.5%

Table: Power to detect an odds ratio of a given magnitude in the rate of participants experiencing any given solicited AE of a specified severity.

Assumed Odds Ratio (nOPV2/Control)	Assumed Rate in Control Arm (%)	Implied Rate in nOPV2 Arm (%)	Power (%)
1.5	2.5	3.7	12.3
	5	7.3	21.1
	7.5	10.8	29.4
	10	14.3	37
	20	27.3	64.5
	25	33.3	70.2
	30	39.1	74.3
2.0	2.5	4.9	31.9
	5	9.5	61.4
	7.5	14.0	79.3
	10	18.2	89
	20	33.3	98.3
	25	40.0	99.4
	30	46.2	99.6
2.5	2.5	6.0	60.7
	5	11.6	90.8
	7.5	16.9	97.3
	10	21.7	99.6

	20	38.5	>99.9
	25	45.5	>99.9
	30	51.7	>99.9
3.0	2.5	7.1	82.5
	5	13.6	98.4
	7.5	19.6	99.9
	10	25.0	>99.9
	20	42.9	>99.9
	25	50.0	>99.9
	30	56.2	>99.9

Assuming ≤10% dropout by the time of receipt of the 2nd dose, ≥603 participants are anticipated to provide safety data following the 2nd dose. The table below displays the probability of detection of events with a specified true rate, in the 603 nOPV2-dosed participants following a 2nd dose. If no events of a given type are detected among 603 participants, the upper bound of the 95% confidence interval is 0.61%.

Table: Probability of detection (observation of 1 or more such events) of events with a specified true rate, in the 603 nOPV2-dosed participants

Probability of observing at least one event	Minimum Event Rate Required
50%	0.12%
80%	0.27%
90%	0.38%
95%	0.50%
**Probability of observing at least two events**	**Minimum Event Rate Required**
50%	0.28%
80%	0.50%
90%	0.64%
95%	0.78%

Young children

Potential use of nOPV2 in an outbreak setting is likely to encompass predominantly children less than 5 years old. The present study provides substantial safety information in the infant participants enrolled in the lot consistency evaluation. In order to further assess safety and tolerability in children between 1 and 5 years old, additional cohorts will be enrolled, primarily for safety evaluation aimed at detection of uncommon events. With 300 children enrolled, there is a 95% probability that one or more such uncommon and important (e.g., vaccine-related serious AE) events would be detected in this group if the rate was greater than or equal to 1 in 100 participants. The table below displays the probability of

detection (observation of 1 or more such events) of events with a specified true rate, in the nOPV2 arm. If no events of a given type are detected among 300 participants, the upper bound of the 95% confidence interval is 1.2%.

Table: Probability of detection (observation of 1 or more such events) of events with a specified true rate, in the nOPV2 arm

Probability of observing at least one event	Minimum Event Rate Required
50%	0.23%
80%	0.53%
90%	0.76%
95%	1.0%
**Probability of observing at least two events**	**Minimum Event Rate Required**
50%	0.56%
80%	1.0%
90%	1.3%
95%	1.6%

To provide a control arm, children will be randomized to receive either 2 doses of nOPV2 or 2 doses of bOPV. Given the limited size of the group of young children to receive nOPV2 described above, 1:1 randomization of nOPV2:bOPV will be used. This is primarily to enable unexpected uncommon events to be observed with equal probability in each arm, should they be spurious and un-associated with vaccination.[5]

Reactogenicity Evaluation

All children aged 1 to 5 years will contribute reactogenicity data during the 7-day period following each dose administration.

The table below describes the power of the two-sided level $\alpha = 0.05$ Fisher's exact test, obtained via simulation, to detect an odds ratio (in the direction of higher rates for the nOPV2 arm only) of a given magnitude in the rate of participants experiencing any given solicited AE of a specified severity. Power ≥ 90% is available for odds ratios ≥2.0 when the control rate is ≥20%. Power ≥80% is available for odds ratios ≥2.5 when the control rate is ≥5.0%.

Table: Power to detect an odds ratio (in the direction of higher rates for the nOPV2 arm only) of a given magnitude in the rate of participants experiencing any given solicited AE of a specified severity

Assumed Odds Ratio (nOPV2/Control)	Assumed Rate in Control Arm (%)	Implied Rate in nOPV2 Arm (%)	Power (%)
1.5	2.5	3.7	10.4
	5	7.3	18.9
	7.5	10.8	25.9
	10	14.3	32.3
	20	27.3	53.3
	25	33.3	59.1
	30	39.1	63.6
2.0	2.5	4.9	28.3
	5	9.5	50.6
	7.5	14.0	68
	10	18.2	79.5
	20	33.3	94.4
	25	40.0	96.8
	30	46.2	97.8
2.5	2.5	6.0	49.3
	5	11.6	80.9
	7.5	16.9	93.3
	10	21.7	97.7
	20	38.5	>99
	25	45.5	>99
	30	51.7	>99
3.0	2.5	7.1	71.7
	5	13.6	94.3
	7.5	19.6	99
	10	25.0	>99
	20	42.9	>99
	25	50.0	>99
	30	56.2	>99

Secondary Evaluations: Infant Post-2nd-dose immune response

All infants in the reactogenicity cohort will receive a 2nd dose and will contribute post-dose-2 data for both reactogenicity and the anti-polio immune response. Per the above, it is anticipated that:

1. 90% of participants will have baseline SNA titres sufficiently low to enable observation of seroconversion.
2. 10% of participants will drop out for reasons unrelated to vaccination.

3. 80% of the remaining nOPV2-administered participants will seroconvert following the first dose.

Further, it may be assumed that 5% of participants who receive the 2nd dose will not provide a post-2nd-dose serum sample for immunogenicity evaluation. Based on these assumptions, of the 670 participants randomized to the nOPV2-administered reactogenicity cohort which will receive a 2nd dose, approximately $670\times0.9\times0.9\times0.2\times0.95 = 103$ participants will remain in the study without seroconverting following the first dose. In preliminary data from the phase 2 study[2], across dose levels, among participants who contributed the necessary samples, had baseline titres low enough to enable observation of seroconversion, and did not seroconvert following the first dose, ≥50% seroconverted following the second. Assuming a conservative seroconversion rate of 50% among such participants in the present study, the expected width of the two-sided 95% exact confidence interval, as an indicator of precision of the estimate, will be approximately ±10%; precision increases if the seroconversion rate is either lower or higher than 50%.

Appendix: Analysis populations

Primary immunogenicity analysis was conducted in the per protocol population which included participants who were randomized, correctly received all study vaccines, had no major protocol deviations considered to potentially interfere with the immunogenicity results on blinded data review, and who had baseline and post-vaccination immunogenicity results available. A supportive assessment in the full analysis population, which did not exclude any participants with protocol deviations, was also performed.

All safety analysis took place in the safety population which included all participants who received at least one dose of a study vaccine. Safety events are presented according to the vaccine received. Solicited adverse events are reported in the reactogenicity population which was a subset of the safety population and consisted of all infants randomized to receive two vaccine doses and all young children.

The viral shedding population included a subset of infants who were randomized to receive a single vaccine dose, had not received bOPV in the preceding two months, and who were negative for type 2 poliovirus at baseline.

Supplementary table: baseline demographic and anthropometric characteristics in infants enrolled in the safety population according to lot received.

	**nOPV2 Lot 1**			**nOPV2 Lot 2**			**nOPV2 Lot 3**		
	**1-Dose Group (N=441)**	**2-Dose Group (N=227)**	**Total (N=668)**	**1-Dose Group (N=441)**	**2-Dose Group (N=227)**	**Total (N=668)**	**1-Dose Group (N=443)**	**2-Dose Group (N=228)**	**Total (N=671)**
	**n (%)**	**n (%)**	**n (%)**	**n (%)**	**n (%)**	**n (%)**	**n (%)**	**n (%)**	**n (%)**
**Sex**									
Female	222 (50.3)	117 (51.5)	339 (50.7)	207 (46.9)	117 (51.5)	324 (48.5)	230 (51.9)	104 (45.6)	334 (49.8)
Male	219 (49.7)	110 (48.5)	329 (49.3)	234 (53.1)	110 (48.5)	344 (51.5)	213 (48.1)	124 (54.4)	337 (50.2)
**Race**									
Black African	440 (99.8)	227 (100.0)	667 (99.9)	440 (99.8)	227 (100.0)	667 (99.9)	442 (99.8)	228 (100.0)	670 (99.9)
Other	1 (0.2)	-	1 (0.1)	1 (0.2)	-	1 (0.1)	1 (0.2)	-	1 (0.1)
**Ethnicity**									
Mandinka	271 (61.5)	112 (49.3)	383 (57.3)	237 (53.7)	147 (64.8)	384 (57.5)	242 (54.6)	130 (57.0)	372 (55.4)
Wolof	32 (7.3)	16 (7.0)	48 (7.2)	42 (9.5)	15 (6.6)	57 (8.5)	33 (7.4)	17 (7.5)	50 (7.5)
Fula	40 (9.1)	35 (15.4)	75 (11.2)	52 (11.8)	18 (7.9)	70 (10.5)	55 (12.4)	27 (11.8)	82 (12.2)
Jola	47 (10.7)	28 (12.3)	75 (11.2)	59 (13.4)	21 (9.3)	80 (12.0)	48 (10.8)	24 (10.5)	72 (10.7)
Other	51 (11.6)	36 (15.9)	87 (13.0)	51 (11.6)	26 (11.5)	77 (11.5)	65 (14.7)	30 (13.2)	95 (14.2)
**Weight for height z-score**									
≥ -2 SD	406 (92.1)	206 (90.7)	612 (91.6)	395 (89.6)	210 (92.5)	605 (90.6)	401 (90.5)	210 (92.1)	611 (91.1)
< -2 SD‡	35 (7.9)	21 (9.3)	56 (8.4)	46 (10.4)	17 (7.5)	63 (9.4)	42 (9.5)	18 (7.9)	60 (8.9)
**Age at screening**									
Median<br>(Min to Max)	33 weeks<br>(19 to 51)	32 weeks<br>(21 to 51)	33 weeks<br>(19 to 51)	33 weeks<br>(18 to 52)	32 weeks<br>(20 to 51)	33 weeks<br>(18 to 52)	32 weeks<br>(20 to 51)	32 weeks<br>(22 to 51)	32 weeks<br>(20 to 51)

N – number of participants included in the analysis; n – number of participants meeting given criterion; % - n/N; SD – standard deviation; Min – minimum value; Max – maximum value; ‡ indicating at least moderate malnutrition.

Supplementary table: prior polio vaccination history in infants and young children – safety population.

		Infants 1-Dose Groups		Infants 2-Dose Groups		Infants Total		Young Children	
		**nOPV2, Any Lot (N=1325)**	**bOPV (N=236)**	**nOPV2, Any Lot (N=682)**	**bOPV (N=102)**	**nOPV2, Any Lot (N=2007)**	**bOPV (N=338)**	**nOPV2 (N=300)**	**bOPV (N=300)**
**Polio Vaccinations Received**		**n (%)**	**n (%)**	**n (%)**	**n (%)**	**n (%)**	**n (%)**	**n (%)**	**n (%)**
**Prior Primary Poliovirus Vaccinations**									
Birth bOPV dose	n (%)	1311 (98.9)	233 (98.7)	670 (98.2)	100 (98.0)	1981 (98.7)	333 (98.5)	NA	NA
1st bOPV dose	n (%)	1325 (100)	236 (100)	682 (100)	102 (100)	2007 (100)	338 (100)	NA	NA
2nd bOPV dose	n (%)	1325 (100)	236 (100)	682 (100)	102 (100)	2007 (100)	338 (100)	NA	NA
3rd bOPV dose	n (%)	1324 (99.9)	236 (100)	682 (100)	102 (100)	2006 (100)	338 (100)	NA	NA
IPV	n (%)	1325 (100)	236 (100)	682 (100)	102 (100)	2007 (100)	338 (100)	NA	NA
**Other Vaccinations**									
Prior IPV†	n (%)	-	1 (0.4)	3 (0.4)	-	3 (0.1)	1 (0.3)	300 (100)	300 (100)
	Mean Number	-	1.0	1.0	-	1.0	1.0	1.0	1.0
	Min, Max	-	1,1	1,1	-	1,1	1,1	1,2	1,2
Prior bOPV§	n (%)	250 (18.9)	29 (12.3)	97 (14.2)	15 (14.7)	347 (17.3)	44 (13.0)	300 (100)	300 (100)
	Mean Number	1.0	1.0	1.0	1.0	1.0	1.0	5.3	5.2
	Min, Max	1,1	1,1	1,1	1,1	1,1	1,1	2,7	2,6

N - Number of participants included in the analysis; n – number of participants meeting given criterion; % - n/N; N/A – not available (data not collected); IPV – inactivated polio vaccine; bOPV – bivalent oral polio vaccine; † for infants count is those >1 prior IPV dose, for young children, any IPV; § for infants, count is those with >4 bOPV, for young children, any bOPV; min – minimum number of doses, max – maximum number of doses; no participant had received any type 2 containing oral polio vaccines (nOPV2, mOPV2 or tOPV)

Supplementary figure: poliovirus type 2 lot to lot equivalence figure – one dose infant per protocol population

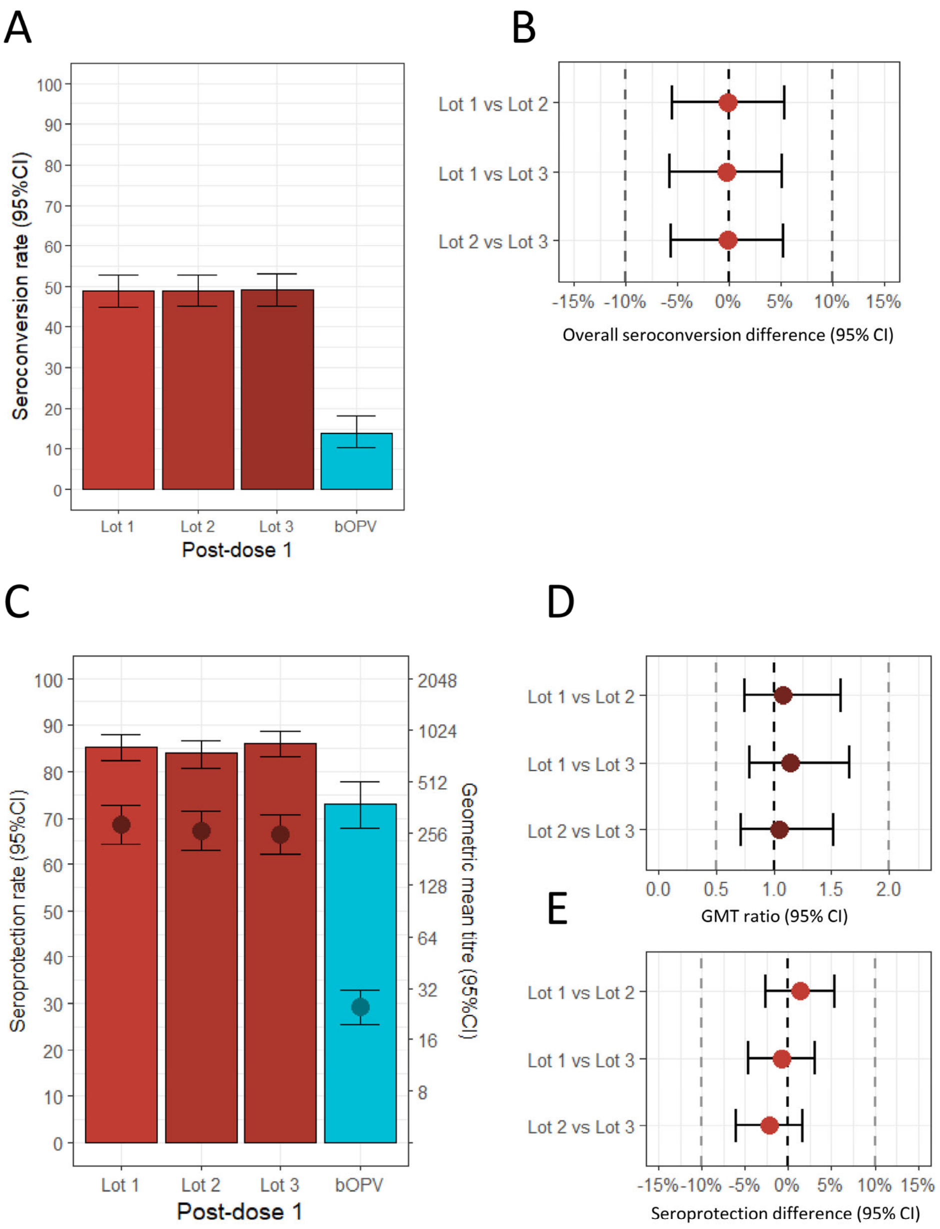

CI – confidence interval; A - overall post-dose 1 poliovirus type 2 serum neutralizing antibody seroconversion rates and 95% CI according to nOPV2 lot or bOPV administered; B – pairwise difference in seroconversion rates between nOPV2 lots with 95% CI (primary lot-to-lot equivalence analysis); C – post-dose 1 poliovirus type 2 serum neutralizing antibody seroprotection rates (solid bars) and geometric mean antibody titres (points) with 95% CI; D – pairwise geometric mean titre ratios between nOPV2 lots with 95% CI (secondary lot-to-lot equivalence analysis); E - pairwise difference in seroprotection rates between nOPV2 lots with 95% CI (secondary lot-to-lot equivalence analysis). Vertical dashed (B, D and E) represent the pre-specified equivalence margins; seroconversion is defined as either a post-vaccination type 2 reciprocal neutralizing antibody titre of ≥ 8 in those infants who were seronegative at baseline (type 2 reciprocal neutralizing antibody titre of < 8) or a four-fold rise in post-vaccination type 2 reciprocal neutralizing antibody titres in infants who were seropositive at baseline including only those infants in which a four-fold rise from baseline was possible to observe based on the upper limit of quantification of the assay; seroprotection is defined as a type 2 reciprocal neutralizing antibody titre of ≥ 8 measured, for the purposes of this analysis, 28 days post-vaccination; ¶ Exact Clopper Pearson 95% confidence intervals around seroconversion and seroprotection rates. Two-sided Miettinen and Nurminen 95% confidence interval around differences in seroconversion and seroprotection rates; † Based on maximum likelihood parameter estimates from a linear model of $\log_2$ serum neutralizing antibody titre as a function of nOPV2 lot arm with a fixed parameter for study clinic and a covariate for the baseline $\log_2$ serum neutralizing antibody titre.

Supplementary table: poliovirus type 2 lot-to-lot seroprotection and geometric mean titre equivalence – one dose infant per protocol population.

		**nOPV2**			**Pairwise Comparisonsβ**		
		**Lot 1**	**Lot 2**	**Lot 3**	**Lot 1 vs Lot 2**	**Lot 1 vs Lot 3**	**Lot 2 vs Lot 3**
Seroprotection rate‡	N	646	653	647			
	n (%)	551 (85.3)	548 (83.9)	557 (86.1)	1.4	-0.8	-2.2
	Exact 95% CI¶	(82.3 to 87.9)	(80.9 to 86.7)	(83.2 to 88.7)	(-2.6 to 5.3)	(-4.6 to 3.0)	(-6.1 to 1.7)
Geometric mean titre†	N	646	653	647			
	GMT†	288.4	265.1	253.2	1.1	1.1	1.0
	95% CI†	(221.1 to 376.2)	(203.3 to 345.8)	(194.1 to 330.2)	(0.75 to 1.58)	(0.79 to 1.65)	(0.72 to 1.52)

N = Number of participants included in the analysis; n = number of participants meeting the definitions for seroprotection; % - n/N; CI – confidence interval; ‡ secondary lot-to-lot equivalence endpoint: seroprotection is defined as a type 2 reciprocal neutralizing antibody titre of ≥ 8 measured, for the purposes of this analysis, 28 days post-vaccination; ¶ Exact Clopper Pearson 95% confidence intervals around seroprotection rates. Two-sides Miettinen and Nurminen 95% confidence interval around differences in seroprotection rates; † Maximum likelihood parameter estimates. Tests are based on a linear model of log_2 NAb titer as a function of nOPV2 lot arm with a fixed parameter for study clinic and a covariate for the baseline log_2 NAb titer level; β Lot-to-lot equivalence was demonstrated based on seroprotection rates if the 95% confidence intervals around the difference between all pairwise lot-to-lot comparisons was contained within the -10% to 10% equivalence interval; lot-to-lot equivalence was demonstrated based on type 2 reciprocal neutralizing antibody titres if the 95% confidence intervals around the pairwise lot-to-lot geometric mean titre ratios was contained within the 0.5 to 2.0 equivalence interval.

Supplementary figure: poliovirus type 2 serum neutralizing antibody reverse cumulative distribution curves in infants and children at the indicated timepoints.

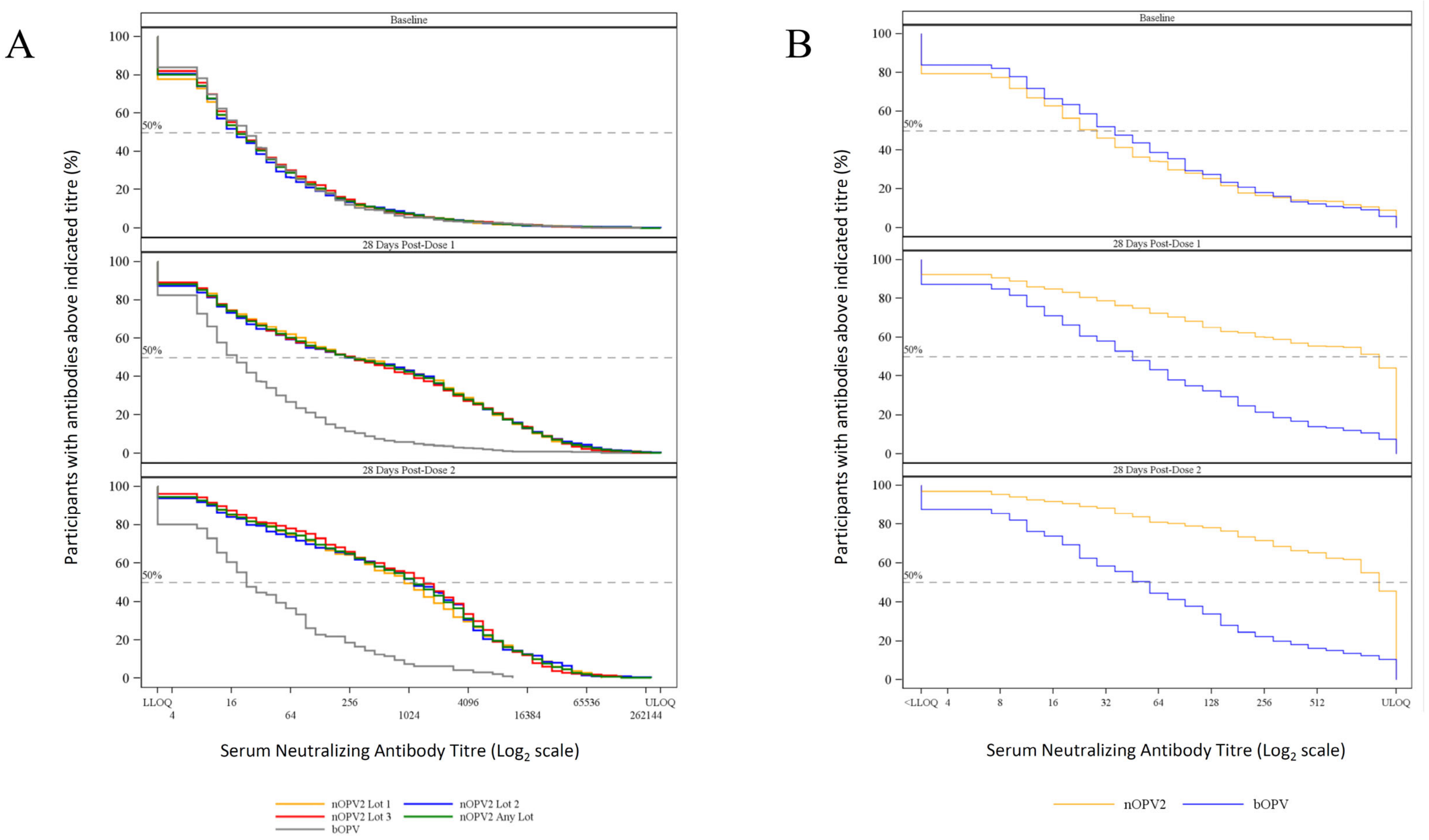

A – infants; B – young children; LLOQ – lower limit of quantification; ULOQ – upper limit of quantification

Supplementary table: solicited adverse events collected between the day of vaccination and day seven after vaccination in the infant and young children reactogenicity cohorts.

	**Infants**			**Young Children**		
	**nOPV2 (N=682)**	**bOPV (N=102)**		**nOPV2 (N=300)**	**bOPV (N=300)**	
**Severity**	**n (%) 95% CI**	**n (%) 95% CI**		**n (%) 95% CI**	**n (%) 95% CI**	
**Any Reaction§**						
Any Severity	273 (40.0) (36.3 to 43.8)	47 (46.1) (36.2 to 56.2)		98 (32.7) (27.4 to 38.3)	98 (32.7) (27.4 to 38.3)	
Severe	0 (0.0) (0.0 to 0.5)	1 (1.0) (0.0 to 5.3)		4 (1.3) (0.4 to 3.4)	2 (0.7) (0.1 to 2.4)	
**Fever‡**						
Any Severity	105 (15.4) (12.8 to 18.3)	26 (25.5) (17.4 to 35.1)	¶	35 (11.7) (8.3 to 15.9)	38 (12.7) (9.1 to 17.0)	
Severe	0 (0.0) (0.0 to 0.5)	1 (1.0) (0.0 to 5.3)		4 (1.3) (0.4 to 3.4)	1 (0.3) (0.0 to 1.8)	
**Vomiting**						
Any Severity	76 (11.1) (8.9 to 13.8)	9 (8.8) (4.1 to 16.1)		15 (5.0) (2.8 to 8.1)	18 (6.0) (3.6 to 9.3)	
Severe	0 (0.0) (0.0 to 0.5)	0 (0.0) (0.0 to 3.6)		0 (0.0) (0.0 to 1.2)	1 (0.3) (0.0 to 1.8)	
**Diarrhoea**						
Any Severity	165 (24.2) (21.0 to 27.6)	25 (24.5) (16.5 to 34.0)		63 (21.0) (16.5 to 26.1)	60 (20.0) (15.6 to 25.0)	
Severe	0 (0.0) (0.0 to 0.5)	0 (0.0) (0.0 to 3.6)		0 (0.0) (0.0 to 1.2)	0 (0.0) (0.0 to 1.2)	
**Irritability**						
Any Severity	40 (5.9) (4.2 to 7.9)	6 (5.9) (2.2 to 12.4)		11 (3.7) (1.8 to 6.4)	4 (1.3) (0.4 to 3.4)	
Severe	0 (0.0) (0.0 to 0.5)	0 (0.0) (0.0 to 3.6)		0 (0.0) (0.0 to 1.2)	0 (0.0) (0.0 to 1.2)	
**Decreased Feeding**						
Any Severity	44 (6.5) (4.7 to 8.6)	10 (9.8) (4.8 to 17.3)		16 (5.3) (3.1 to 8.5)	23 (7.7) (4.9 to 11.3)	
Severe	0 (0.0) (0.0 to 0.5)	0 (0.0) (0.0 to 3.6)		0 (0.0) (0.0 to 1.2)	0 (0.0) (0.0 to 1.2)	
**Decreased Activity**						
Any Severity	26 (3.8) (2.5 to 5.5)	4 (3.9) (1.1 to 9.7)		9 (3.0) (1.4 to 5.6)	10 (3.3) (1.6 to 6.0)	
Severe	0 (0.0) (0.0 to 0.5)	0 (0.0) (0.0 to 3.6)		0 (0.0) (0.0 to 1.2)	0 (0.0) (0.0 to 1.2)	

N - number of participants in reactogenicity cohort; n - number of participants experiencing given event; % - n/N;Each participant is only counted once per event based on the maximum reported severity during the seven day solicitation period; CI – confidence interval; 95% confidence intervals calculated using the exact Clopper Pearson method; § Fever (axillary temperature ≥ 37.5°C), vomiting, diarrhoea, irritability, decreased feeding, decreased activity [including immediate solicited events occurring at 30 minutes post-vaccination in the reactogenicity population]; ‡ defined as an axillary temperature of ≥ 37.5°C; ¶ p = 0.015 – Fisher's exact two-tailed test of the rate of events between nOPV2 and bOPV; no other difference in event rate between nOPV2 and bOPV within age cohorts significant at the 5% significance level.

Supplementary table: unsolicited adverse events occurring in ≥2% of infants.

	nOPV2 (N = 2007)		bOPV (N = 338)	
**Adverse event§**	**Number of Events**	**n (%) 95% CI**	**Number of Events**	**n (%) 95% CI**
**Upper respiratory tract infection**				
Any Severity	337	327 (16.3) (14.7 to 18.0)	57	57 (16.9) (13.0 to 21.3)
Severe	0	0 (0.0) (0.0 to 0.2)	0	0 (0.0) (0.0 to 1.1)
**Gastroenteritis**				
Any Severity	137	131 (6.5) (5.5 to 7.7)	31	31 (9.2) (6.3 to 12.8)
Severe	2	2 (0.1) (0.0 to 0.4)	1	1 (0.3) (0.0 to 1.6)
**Diarrhoea**				
Any Severity	80	78 (3.9) (3.1 to 4.8)	15	15 (4.4) (2.5 to 7.2)
Severe	0	0 (0.0) (0.0 to 0.2)	0	0 (0.0) (0.0 to 1.1)
**Conjunctivitis**				
Any Severity	60	60 (3.0) (2.3 to 3.8)	12	12 (3.6) (1.9 to 6.1)
Severe	0	0 (0.0) (0.0 to 0.2)	0	0 (0.0) (0.0 to 1.1)
**Furuncle**				
Any Severity	56	55 (2.7) (2.1 to 3.6)	11	10 (3.0) (1.4 to 5.4)
Severe	0	0 (0.0) (0.0 to 0.2)	0	0 (0.0) (0.0 to 1.1)
**Lower respiratory tract infection**				
Any Severity	39	39 (1.9) (1.4 to 2.7)	9	9 (2.7) (1.2 to 5.0)
Severe	2	2 (0.1) (0.0 to 0.4)	1	1 (0.3) (0.0 to 1.6)
**Bronchiolitis**				
Any Severity	35	34 (1.7) (1.2 to 2.4)	13	12 (3.6) (1.9 to 6.1)
Severe	5	5 (0.2) (0.1 to 0.6)	1	1 (0.3) (0.0 to 1.6)

N – number in cohort (safety population); n – number of participants experiencing a given event based on the maximum severity of the event throughout its course; % - n/N; CI – confidence interval; § MedDRA preferred term; includes all unsolicited AE reported within 28 days of vaccine administration and any unsolicited AE also associated with serious adverse events throughout the trial except if occurring after a dose of nOPV2 administered during an outbreak-response campaign; 95% confidence intervals calculated using the exact Clopper Pearson method.

Supplementary table: unsolicited adverse events occurring in ≥2% of young children.

	nOPV2 (N = 300)		bOPV (N = 300)	
**Adverse event§**	**Number of Events**	**n (%) 95% CI**	**Number of Events**	**n (%) 95% CI**
**Upper respiratory tract infection**				
Any Severity	73	64 (21.3) (16.8 to 26.4)	66	60 (20.0) (15.6 to 25.0)
Severe	0	0 (0.0) (0.0 to 1.2)	0	0 (0.0) (0.0 to 1.2)
**Gastroenteritis**				
Any Severity	20	20 (6.7) (4.1 to 10.1)	27	26 (8.7) (5.7 to 12.4)
Severe	0	0 (0.0) (0.0 to 1.2)	0	0 (0.0) (0.0 to 1.2)
**Furuncle**				
Any Severity	17	15 (5.0) (2.8 to 8.1)	11	11 (3.7) (1.8 to 6.5)
Severe	0	0 (0.0) (0.0 to 1.2)	0	0 (0.0) (0.0 to 1.2)
**Lower respiratory tract infection**				
Any Severity	12	12 (4.0) (2.1 to 6.9)	12	12 (4.0) (2.1 to 6.9)
Severe	0	0 (0.0) (0.0 to 1.2)	0	0 (0.0) (0.0 to 1.2)
**Impetigo**				
Any Severity	12	12 (4.0) (2.1 to 6.9)	10	10 (3.3) (1.6 to 6.0)
Severe	0	0 (0.0) (0.0 to 1.2)	0	0 (0.0) (0.0 to 1.2)
**Diarrhoea**				
Any Severity	11	11 (3.7) (1.8 to 6.5)	9	8 (2.7) (1.2 to 5.2)
Severe	0	0 (0.0) (0.0 to 1.2)	0	0 (0.0) (0.0 to 1.2)
**Conjunctivitis**				
Any Severity	8	8 (2.7) (1.2 to 5.2)	11	10 (3.3) (1.6 to 6.0)
Severe	0	0 (0.0) (0.0 to 1.2)	0	0 (0.0) (0.0 to 1.2)
**Tinea capitis**				
Any Severity	11	11 (3.7) (1.8 to 6.5)	8	7 (2.3) (0.9 to 4.8)
Severe	0	0 (0.0) (0.0 to 1.2)	0	0 (0.0) (0.0 to 1.2)
**Body tinea**				
Any Severity	3	3 (1.0) (0.2 to 2.9)	9	9 (3.0) (1.4 to 5.6)
Severe	0	0 (0.0) (0.0 to 1.2)	0	0 (0.0) (0.0 to 1.2)
**Thermal burn**				
Any Severity	6	6 (2.0) (0.7 to 4.3)	4	4 (1.3) (0.4 to 3.4)
Severe	0	0 (0.0) (0.0 to 1.2)	0	0 (0.0) (0.0 to 1.2)

N – number in cohort (safety population); n – number of participants experiencing a given event based on the maximum severity of the event throughout its course; % - n/N; CI – confidence interval; § MedDRA preferred term; includes all unsolicited AE reported within 28 days of vaccine administration and any unsolicited AE also associated with serious adverse events throughout the trial except if occurring after a dose of nOPV2 administered during an outbreak-response campaign; 95% confidence intervals calculated using the exact Clopper Pearson method.

Supplementary table: serious adverse event listing – infants and young children

**Diagnosis§**		**Group**	**Last dose**	**SAE designation**	**Onset day post-vaccination**	**SAE duration**	**Severity**	**Related**	**Outcome**
Gastroenteritis	nOPV2	Young child – 2 dose	1	Hospitalization	15	9	Moderate	No	Recovered
Vomiting	bOPV	Young child – 2 dose	1	Hospitalization	3	15	Severe	Yes	Recovered
Pneumonia	bOPV	Young child – 2 dose	2	Hospitalization	47	6	Moderate	No	Recovered
Bronchiolitis	bOPV	Young child – 2 dose	2	Hospitalization	23	5	Severe	No	Recovered
Gastroenteritis	bOPV	Young child – 2 dose	2	Hospitalization	21	5	Moderate	No	Recovered
Gastroenteritis	nOPV2	Infant – 1 dose	1	Hospitalization	13	3	Moderate	No	Recovered
Gastroenteritis	nOPV2	Infant – 2 dose	1	Hospitalization	2	6	Moderate	No	Recovered
Meningitis pneumococcal	nOPV2	Infant – 2 dose	2	Hospitalization	68	27	Severe	No	Recovered
Bronchiolitis	nOPV2	Infant – 2 dose	2	Hospitalization	38	5	Severe	No	Recovered
Lower respiratory tract infection	nOPV2	Infant – 1 dose	1	Hospitalization	58	9	Severe	No	Recovered
Lower respiratory tract infection	nOPV2	Infant – 2 dose	2	Hospitalization	14	2	Moderate	No	Recovered
Bronchiolitis	bOPV	Infant – 1 dose	1	Hospitalization	38	10	Severe	No	Recovered
Peri-orbital cellulitis	nOPV2	Infant – 1 dose	1	Hospitalization	83	6	Severe	No	Recovered
Bronchiolitis	nOPV2	Infant – 2 dose	1	Hospitalization	32	8	Moderate	No	Recovered
Sepsis	bOPV	Infant – 2 dose	2	Hospitalization – Death	54	4	Fatal	No	Died
Bronchiolitis‡	nOPV2	Infant – 1 dose	1	Hospitalization	11	4	Severe	No	Recovered
Thermal burn‡	nOPV2	Infant – 1 dose	1	Hospitalization	83	Ongoing†	Severe	No	Ongoing
Lower respiratory tract infection	nOPV2	Infant – 2 dose	2	Hospitalization	27	4	Severe	No	Recovered
Accident (fire-related injury¶)	nOPV2	Infant – 1 dose	1	Death	109	1	Fatal	No	Died
Gastroenteritis	nOPV2	Infant – 2 dose	2	Hospitalization	57	15	Severe	No	Recovered
Gastroenteritis	nOPV2	Infant – 2 dose	2	Hospitalization	15	9	Severe	No	Recovered

**Diagnosis§**		**Group**	**Last dose**	**SAE designation**	**Onset day post-vaccination**	**SAE duration**	**Severity**	**Related**	**Outcome**
Bronchiolitis	nOPV2	Infant – 1 dose	1	Hospitalization	61	6	Severe	No	Recovered
Bronchiolitis	nOPV2	Infant – 1 dose	1	Hospitalization	33	4	Severe	No	Recovered
Bronchiolitis	nOPV2	Infant – 2 dose	1	Hospitalization	6	3	Severe	No	Recovered
Bronchiolitis	bOPV	Infant – 1 dose	1	Hospitalization	8	9	Moderate	No	Recovered
Gastroenteritis	bOPV	Infant – 2 dose	1	Hospitalization	19	3	Severe	No	Recovered
Lower respiratory tract infection	bOPV	Infant – 1 dose	1	Hospitalization	16	7	Severe	No	Recovered
Pneumonia	nOPV2	Infant – 1 dose	1	Hospitalization	67	4	Severe	No	Recovered

§ MedDRA preferred term; ‡ Two SAEs in the same participant; † ongoing at end of study visit; ¶ verbatim term reported.

Supplementary table: poliovirus shedding rates among infants.

	Time with relation to vaccine dose ¶			
	Baseline§	Day 8	Day 29	Day 85
Serotype	n/N (%)	n/N (%)<br>95% CI	n/N (%)<br>95% CI	n/N (%)<br>95% CI
**nOPV2**				
Type 1	2/251 (0.8)	2/187 (1.1)<br>(0.1 to 3.8)	0/186 (0.0)<br>(0.0 to 2.0)	0/112 (0.0)<br>(0.0 to 3.2)
Type 2	5/251 (2.0)	78/187 (41.7)<br>(34.6 to 49.1)	8/186 (4.3)<br>(1.9 to 8.3)	0/112 (0.0)<br>(0.0 to 3.2)
Type 3	3/251 (1.2)	3/187 (1.6)<br>(0.3 to 4.6)	2/186 (1.1)<br>(0.1 to 3.8)	0/112 (0.0)<br>(0.0 to 3.2)
**bOPV**				
Type 1	1/42 (2.4)	7/34 (20.6)<br>(8.7 to 37.9)	0/32 (0.0)<br>(0.0 to 10.9)	0/22 (0.0)<br>(0.0 to 15.4)
Type 2	0/42 (0.0)	1/34 (2.9)<br>(0.1 to 15.3)	0/32 (0.0)<br>(0.0 to 10.9)	0/22 (0.0)<br>(0.0 to 15.4)
Type 3	1/42 (2.4)	9/34 (26.5)<br>(12.9 to 44.4)	0/32 (0.0)<br>(0.0 to 10.9)	0/22 (0.0)<br>(0.0 to 15.4)

§ Includes all infants providing a pre-vaccination stool sample; ¶ Includes infants in the Viral Shedding Population, which required no bOPV receipt 2 months prior to first study vaccination, randomization into the cohort, and a pre-vaccination stool sample negative for type 2 virus.

Supplementary table: cessation of poliovirus type 2 viral shedding following a single nOPV2 dose.

	Positivity defined by RT-PCR	
	Number of infants	Percentage of participants shedding*<br>(95% CI)
**Day 7**	187	41.5%<br>(34.5 – 48.3)
**Day 28**	186	4.5%<br>(2.2 – 7.8)
**Day 84**	112	0.0%

* Survival function estimates derived from SAS procedure ICLIFETEST, using the expectation-maximization iterative convex minorant algorithm.

REFERENCES

1. Konz JO, Schofield T, Carlyle S, et al. Evaluation and validation of next-generation sequencing to support lot release for a novel type 2 oral poliovirus vaccine. *Vaccine: X* 2021; 8: 100102.
2. Saez-Llorens X, Bandyopadhyay AS, Gast C, et al. Safety and immunogenicity of two novel type 2 oral poliovirus vaccine candidates compared with a monovalent type 2 oral poliovirus vaccine in children and infants: two clinical trials. *Lancet* 2021; 397(10268): 27-38.
3. Miettinen O, Nurminen M. Comparative analysis of two rates. *Statistics in medicine* 1985; 4(2): 213-26.
4. Fagerland MW, Lydersen S, Laake P. Recommended confidence intervals for two independent binomial proportions. *Statistical methods in medical research* 2015; 24(2): 224-54.
5. CIOMS. Guidelines for Preparing Core Clinical-Safety Information on Drugs; 1999.
